# A scoping review on emerging biomarkers in inflammatory bowel disease: Towards precision medicine in diagnosis and therapeutic management

**DOI:** 10.1371/journal.pone.0353295

**Published:** 2026-07-13

**Authors:** Matheus Querino da Silva, João Daniel de Souza Menezes, José Luis Esteves Francisco, Franciane Michele da Silva, Paula Camile Vieira Rocha, Paula Buck de Oliveira Ruiz, Caroline Cristina dos Santos Costa, Laís Fernanda de Amorin, Clarissa Albuquerque Vaz Nunes, Nilton Sebastião Garcia de Almeida Neto, Lorena Aline dos Santos, Guilherme Ribeiro Constancio, Maira Ceruti Mendes, Ana Paula Bernardes da Rosa, Natália Ferreira Rondina, Salvador André Bavaresco Cristovão, Marcia Regina Furlani, Janaína Aparecida de Sales Floriano, Emerson Roberto dos Santos, Fernando Nestor Facio Júnior, Luís Cesar Fava Spessoto, Rita de Cássia Helú Mendonça Ribeiro, Maysa Alahmar Bianchin, Marco Antônio Ribeiro Filho, Marli de Carvalho Jericó, Antônio Hélio Oliani, Denise Cristina Móz Vaz Oliani, Nádia Antônia Aparecida Poletti, Mikaell Alexandre Gouvea Faria, Júlio César André

**Affiliations:** 1 Center for Studies and Development of Health Education – CEDES, São José do Rio Preto Medical School - FAMERP, Avenida Brigadeiro Faria Lima, São José do Rio Preto, São Paulo, Brazil; 2 Faculdade de Medicina de Ribeirão Preto - USP, Ribeirão Preto, São Paulo, Brazil; 3 Expedicionários da Saúde, Sousas, Avenida Dona Maria Franco Salgado, Jardim Atibaia (Sousas), Campinas, São Paulo, Brazil; 4 Hospital de Amor Amazônia, Brazil; 5 Centro Universitário do Norte de São Paulo, São José do Rio Preto, São Paulo, Brazil; 6 Faculdade de Medicina de Catanduva, Catanduva, São Paulo, Brazil; 7 Real e Benemérita Associação Portuguesa de Beneficência, São Paulo, São Paulo, Brazil; 8 University of Santo Amaro - UNISA, Santo Amaro, São Paulo, Brazil; 9 University Hospital Center Cova da Beira, University of Beira Interior, Covilhã, Portugal; GLA University, INDIA

## Abstract

**Background:**

Inflammatory Bowel Diseases (IBD) are chronic conditions presenting significant diagnostic and management challenges. Current invasive methods and traditional biomarkers often lack sufficient accuracy and fail to address the disease’s heterogeneity and unpredictable therapeutic responses. This necessitates more precise, personalized clinical tools.

**Methods:**

This scoping review synthesized recent findings on emerging biomarkers for IBD. We focused on technological advances in omics platforms (genomics, transcriptomics, proteomics, metabolomics, microbiomics), artificial intelligence, biosensors, and imaging techniques. Analysis identified biomarker potential for early diagnosis, disease activity monitoring, progression prognosis, and therapeutic response prediction. The review adhered to PRISMA-ScR guidelines and was registered with the Open Science Framework (OSF). The search encompassed five major databases: PubMed/MEDLINE, Scopus, Web of Science, Embase, and Google Scholar.

**Results:**

Studies demonstrate vast potential in non-invasive biomarkers for refined early diagnosis, optimized disease monitoring, and treatment response prediction. Key findings include metabolomic and gut microbiota profiles, genetic and epigenetic markers, and AI integration of complex data. These approaches promise to overcome conventional indicator limitations. From 784 initial records, 27 articles were included, published between 2021 and 2025.

**Conclusion:**

Emerging biomarkers are fundamental for the transition to precision medicine in IBD. Their implementation aims to enhance pathogenesis understanding, personalize therapies, and improve patient quality of life, establishing pathways for more effective, individualized management approaches.

## 1. Introduction

Inflammatory Bowel Diseases (IBD), encompassing Crohn’s Disease (CD) and Ulcerative Colitis (UC), are chronic, debilitating conditions affecting millions globally [1, 2]. Characterized by persistent gastrointestinal tract inflammation with periods of remission and exacerbation [3], IBD has a multifactorial etiology. This involves a complex interplay among genetic factors [4], environmental influences, gut microbiota dysbiosis [5], and immune system dysregulation. The increasing prevalence, particularly in pediatric populations [6], and the significant impact on patient quality of life and healthcare systems, make IBD management a continuous challenge.

Historically, IBD diagnosis and monitoring relied on invasive methods such as endoscopy and biopsies. While considered the “gold standard,” these procedures are costly, uncomfortable, and carry risks [7]. Furthermore, conventional biomarkers like C-reactive protein (CRP) and fecal calprotectin (FC), though useful, have limitations. These include non-specificity in certain situations or difficulty in accurately reflecting mucosal inflammation severity. The heterogeneous nature of IBD, varying among patients, and the unpredictable response to available treatments underscore the urgent need for more precise and personalized tools. These tools should optimize clinical decision-making, from evaluating general biomarkers [8] to predicting therapeutic response [9].

Against this background, research into emerging biomarkers has experienced exponential growth, driven by advancements in omics technologies (genomics, transcriptomics, proteomics, metabolomics, microbiomics) and artificial intelligence, including Machine Learning (ML) approaches. These novel biomarkers promise to overcome existing limitations by offering less invasive methods with greater diagnostic and prognostic accuracy, as well as the ability to predict therapeutic response. Investigations range from biomarker profiles related to oxidative stress and adiponectin ([10] to the evaluation of cellular ratios such as NLR, LMR, and PLR [11]. The ultimate goal is to transition from a “one-size-fits-all” model to precision medicine. This approach tailors treatment to each patient’s individual biological characteristics, including identifying preclinical protein signatures [12] and potential new therapeutic targets like galectin-3 [13] and formyl peptide receptor 2 [14].

Given the rapid increase in studies exploring a wide range of biomarkers—from metabolomic and gut microbiota profiles to genetic, epigenetic, and protein markers, alongside the potential of imaging technologies and biosensors ([15, 16] and considering the urgent need for more effective IBD management tools, this review was developed. Its objective is to synthesize and correlate the most recent findings on emerging biomarkers in the diagnosis and evaluation of intestinal inflammatory lesions in IBD. By compiling and critically analyzing this literature, we aim to highlight the potential of these biomarkers to improve the understanding of IBD pathogenesis, refine early diagnosis, optimize monitoring of disease activity, and guide more effective and personalized therapeutic strategies, solidifying the path for future IBD management.

## 2. Methodology

### 2.1. Study design

This scoping review was conducted following the guidelines of the PRISMA Extension for Scoping Reviews (PRISMA-ScR), as established by Tricco et al. (2018) [17]. The review protocol was previously registered with the Open Science Framework (OSF) to ensure transparency and reproducibility of the research.

### 2.2. Protocol Registration

The protocol for this scoping review was registered on the Open Science Framework (OSF) platform before the start of data collection, following best practices in scientific research and ensuring the methodological transparency of the study.

### 2.3. Ethical Aspects

This scoping review utilized only data from public sources and previously published scientific literature. As a literature review, it did not require approval by a Research Ethics Committee, as established by CNS Resolution No. 466/2012. All copyrights were respected, and sources were duly cited according to current academic standards.

### 2.4. Guiding Question

The guiding question for this review was formulated following the PCC (Population, Concept, Context) strategy:

**P (Population):** Patients with inflammatory bowel diseases **C (Concept):** Emerging biomarkers for diagnosis and assessment **C (Context):** Current scientific literature on IBD diagnosis

**Guiding question:** “What is widely reported regarding emerging biomarkers for the diagnosis and assessment of intestinal inflammatory lesions?”

### 2.5. Search Strategy

The search strategy was applied in five major databases: PubMed/MEDLINE, Scopus, Web of Science, Embase, and Google Scholar (Table 1) and the pre-established inclusion and exclusion criteria (Table 2):

**Table 1:** Search Strategy.

Database	Search Strategy
PubMed/MEDLINE	(“inflammatory bowel disease*” OR “IBD” OR “Crohn’s disease” OR “ulcerative colitis”) AND (“biomarker*” OR “biological marker*” OR “molecular marker*” OR “diagnostic marker*”) AND (“diagnosis” OR “diagnostic” OR “assessment” OR “evaluation”)
Scopus	TITLE-ABS-KEY((“inflammatory bowel disease*” OR “IBD” OR “Crohn’s disease” OR “ulcerative colitis”) AND (“biomarker*” OR “biological marker*” OR “molecular marker*”) AND (“diagnosis” OR “assessment”))
Web of Science	TS=((“inflammatory bowel disease*” OR “IBD” OR “Crohn’s disease” OR “ulcerative colitis”) AND (“biomarker*” OR “biological marker*”) AND (“diagnosis” OR “evaluation”))
Embase	(‘inflammatory bowel disease’/exp OR ‘Crohn disease’/exp OR ‘ulcerative colitis’/exp) AND (‘biomarker’/exp OR ‘biological marker’/exp OR ‘molecular marker’/exp OR ‘diagnostic marker’/exp) AND (‘diagnosis’/exp OR ‘assessment’/exp OR ‘evaluation’/exp)
Google Scholar	(“inflammatory bowel disease” OR “IBD” OR “Crohn’s disease” OR “ulcerative colitis”) AND (“biomarker” OR “biological marker” OR “molecular marker” OR “diagnostic marker”) AND (“diagnosis” OR “assessment” OR “evaluation”)

**Table 2:** Eligibility Criteria.

Criterion	Inclusion	Exclusion
Publication Period	Studies published between January 2021 and December 2025.	Articles published before 2021.
Publication Type	Original research articles (clinical trials, randomized controlled trials, cohort studies, case-control studies) and reviews (systematic or narrative).	Letters to the editor, editorials, commentaries, theses, dissertations, and conference abstracts lacking full text.
Languages	English, Portuguese, Spanish.	Other languages.
Primary Thematic Focus	Studies on the identification, characterization, validation, or clinical application of biomarkers specifically for Inflammatory Bowel Disease (IBD).	Studies focused exclusively on treatments, epidemiology, or pathophysiology without exploring the role of biomarkers.
Biomarker Type	Emerging or novel biomarkers (genetic, epigenetic, proteomic, metabolomic, microbiomic, imaging, etc.) or established biomarkers with new applications/perspectives.	–
Biomarker Application	Focus on the applicability of biomarkers for: diagnosis, disease activity assessment, patient stratification, prognosis of progression, prediction of therapeutic response, or detection of complications.	–
Study Population	Studies conducted on human populations (adults and/or pediatric).	Studies conducted exclusively on animal models (in vitro, in silico, non-human animals).
Availability	Full text readily available.	Duplicate studies or articles without full text access.
Study Design	Clinical studies (prospective or retrospective), randomized clinical trials, cohort studies, or case-control studies evaluating biomarker performance.	Purely laboratory studies (e.g., analytical method development without clinical sample validation), purely methodological studies (e.g., equipment calibration), or studies with insufficient data for biomarker performance evaluation.
Performance Metrics	Articles reporting biomarker performance metrics (e.g., sensitivity, specificity, positive/negative predictive value, AUC, likelihood ratio) or correlation analyses with clinical/endoscopic outcomes.	Studies that only describe the presence of a biomarker without any analysis of its diagnostic, prognostic, or predictive capability.
Biological Samples	Studies utilizing biological samples relevant to IBD (e.g., serum, plasma, feces, tissue biopsies, urine, sweat).	–
Comparative Analysis	Studies comparing the performance of novel biomarkers with established markers (e.g., fecal calprotectin, CRP) or among themselves, and those comparing different disease states (active vs. remission, IBD vs. healthy controls, IBD vs. other gastrointestinal conditions).	Studies that do not conduct any biomarker comparison or fail to contextualize the biomarker’s performance relative to disease state or known markers.

### 2.6. Study selection Process

Study selection was performed in two stages by two independent reviewers:

• Initial screening: Analysis of titles and abstracts
• Final selection: Full-text reading of selected articles

In case of disagreement, a third reviewer was consulted for a final decision. Rayyan QCRI software was used for reference management and the selection process.

### 2.7. Data extraction

Data were extracted using a standardized form (Table 3) by two independent reviewers. Discrepancies were resolved by consensus.

**Table 3:** Data Collection Instrument.

Category	Variable	Description
Study Identification	Main author	First author of the study
	Publication year	Year of article publication
	Country	Country where the study was conducted
	Journal	Name of the scientific journal
	Study type	Methodological design (cross-sectional, cohort, case-control, etc.)
	Level of evidence	Classification according to Oxford Centre for Evidence-Based Medicine
Population	Sample size	Total number of participants
	Age (mean ± SD)	Mean age of participants
	Sex (%)	Distribution by sex
	IBD type	Crohn’s Disease, Ulcerative Colitis, unclassified IBD
Biomarkers	Biomarker type	Protein, genetic, metabolic, immunological, etc.
	Biomarker name	Specific name of the studied marker
	Detection method	Laboratory technique used
	Sample type	Serum, plasma, feces, tissue, urine, etc.
	Diagnostic purpose	Initial diagnosis, monitoring, prognosis, disease activity
Results	Sensitivity (%)	Ability to detect true positives
	Specificity (%)	Ability to detect true negatives
	Positive predictive value (%)	Probability of disease when test is positive
	Negative predictive value (%)	Probability of absence of disease when test is negative
Methodological Quality	Control group	Presence and characteristics of the control group
	Blinding	Type of blinding used
	Reported limitations	Methodological limitations reported by the authors

### 2.8. Data synthesis

After a detailed literature search process, pertinent data were systematically extracted. The initial data extraction was performed by two independent reviewers, with the aim of minimizing bias and ensuring the completeness and accuracy of the information. In case of any disagreement between reviewers regarding the inclusion or interpretation of a specific datum, a third reviewer was consulted for adjudication and consensus. Subsequently, the extracted data were narratively synthesized, meticulously organized, and categorized according to the identified biomarker types, their respective diagnostic characteristics, potential clinical applicability at different disease stages, and existing knowledge gaps in the area, guiding future research. A table was created for each selected article responding to the data collection instrument (Tables 2-28 supplementary material in S1 File).

## 3. Results

After applying the search strategy in the PubMed/MEDLINE, Scopus, Web of Science, Embase, and Google Scholar databases, and subsequent removal of duplicates, 406 articles were identified. Of these, 27 articles were selected for this review, strictly adhering to the inclusion and exclusion criteria. The study selection process is illustrated in Image 1.

**Fig 1:**
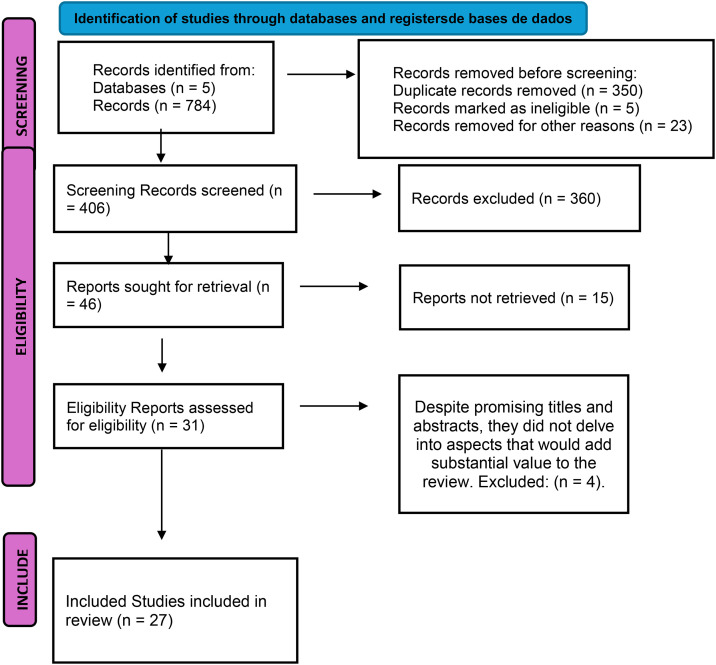
Flowchart of selected articles.

After applying the search strategy across PubMed/MEDLINE, Scopus, Web of Science, Embase, and Google Scholar, and subsequent removal, a total of 406 articles were identified. Of these, 27 articles were selected for this review, strictly adhering to the inclusion and exclusion criteria. The study selection process is illustrated in Fig 1.

The study selection process followed PRISMA guidelines. It began with identifying over 784 records from five databases. After removing duplicates (n = 350), ineligible records (n = 5), and other reasons (n = 23), 406 unique records advanced to screening. Title and abstract evaluation led to the exclusion of 360 records not aligning with the review’s scope, resulting in 46 reports potentially relevant for full-text analysis. Of the 46 reports sought for retrieval, 15 were not included because their full texts were not available for detailed eligibility assessment at the time of screening, despite being identified as potentially relevant records. The remaining 31 reports underwent rigorous full-text eligibility assessment. 4 were excluded for not sufficiently elaborating on aspects of substantial value. Finally, 27 studies were included, forming the basis for data synthesis.

The supplementary material for this article includes a comprehensive overview of the included studies. Table S1 in S1 File provides a general description of all articles encompassed in this review, while Tables S2 to S28 in S1 File offer detailed data extraction for each of the 27 individual articles included in the analysis.

### Description of Selected Studies

The 27 included studies, published between 2021 and 2025, demonstrate continuous interest in identifying and validating IBD biomarkers. The annual distribution shows a significant concentration of publications in 2024 (10 articles, 37.0%) and 2021 (7 articles, 25.9%). Six articles (22.2%) were published or accepted for 2025, and two articles (7.4%) in both 2022 and 2023. Geographically, research is diverse, with contributions from various countries, notably China and the USA.

The methodology varied, ranging from observational cross-sectional and retrospective studies to complex mega-analyses and Mendelian randomization studies. A significant portion (14 articles, 51.9%) comprised comprehensive and narrative reviews. The remaining studies focused primarily on diagnostic accuracy and machine learning, contributing original data on new marker performance. The main target population was adult patients with Crohn’s Disease (CD), Ulcerative Colitis (UC), and IBD generally, though pediatric studies were also included [18, 19]. Sample sizes varied widely, from small groups for proteomic technique validation [20] to large cohorts in population genomic studies [21].

### Key findings on Emerging Biomarkers

Current research on emerging IBD biomarkers is vast and multifaceted, covering various categories that offer new perspectives for diagnosis, monitoring, and prognosis.

### Metabolomic Biomarkers

In metabolomics, fecal nervonic acid emerges as a promising biomarker. Kunst et al. (2024) observed significantly higher levels of nervonic acid in IBD patient feces compared to controls (P < 0.001) [22]. It distinguished groups with an AUROC of 0.827 (P < 0.001), sensitivity of 71%, and specificity of 82% (cutoff 0.49 µmol/g). This marker also correlated positively with serum C-reactive protein (CRP) (r = 0.376, P < 0.01) and fecal calprotectin (FC) (r = 0.575, P < 0.001). It could discriminate patients with FC levels ≥ 120 µg/g with an AUROC of 0.856 (P < 0.001). Reinforcing these findings, Huss et al. (2025) identified arachidonic acid (AA) and adrenic acid (AdA) as elevated in IBD patient feces compared to controls (AA: AUROC 0.859, P < 0.001; AdA: AUROC 0.841, P < 0.001), both correlating positively with FC [23]. Boye et al. (2024) also highlighted metabolomics’ potential to distinguish IBD from non-IBD and its subtypes with AUCs above 0.8, predicting anti-TNF therapy response (fecal lipids AUC = 0.94) [24].

Furthermore, serum lipoprotein levels, including LDL and HDL, were investigated by Zhao et al. (2025) [25]. They found significantly lower concentrations in IBD patients than in controls (P < 0.001 for both). HDL correlated negatively with activity indices such as HBI (r = −0.341, P < 0.001) and CDEIS (r = −0.304, P < 0.001) in CD. For moderate-to-severe UC, LDL showed a sensitivity of 65.65%, and HDL a specificity of 87.34%, outperforming CRP in some aspects.

### Microbiological Biomarkers

The gut microbiota, comprising bacteria, viruses, and fungi, is crucial in IBD pathogenesis. Wang et al. (2024) emphasized dysbiosis as a critical factor and indicated that the microbiota can serve as biomarkers with AUCs up to 0.869 [19]. *Akkermansia muciniphila*, when depleted, was identified as a potential biomarker for pediatric CD. *Faecalibacterium prausnitzii* and *Escherichia coli* were useful in phenotypic categorization of CD (Sn 82.50% for differentiating ileal from colonic CD).

Sharma et al. (2025) explored dysbiosis further, describing reduced microbial diversity and compositional changes, such as a decrease in *F. prausnitzii* and an increase in *E. coli*. Microbial metabolomics revealed reduced SCFAs and increased pro-inflammatory metabolites, highlighting the complex microbiota-host interaction [26].

### Genetic and Epigenetic Biomarkers

Genetic and epigenetic contributions are increasingly recognized. Zhao et al. (2024), through Mendelian randomization, identified 8 gut microbiota species and 9 inflammatory proteins with causal relationships to IBD. Genera such as *Candidatus Soleaferrea* (OR = 1.227, P = 0.001) and *Ruminococcaceae UCG013* (OR = 1.293, P = 0.002) increase IBD risk. *Clostridiaceae1* and *Ruminococcaceae UCG002* are protective. Proteins like IL-10Rα and MMP-10 mediate the effects of *Clostridiaceae1* in IBD (22.73% and 17.61% mediation, respectively) [21].

Xu et al. (2021) and Petito et al. (2024) explored the emerging roles of circRNAs and eccDNA. CircRNAs, with dysfunction linked to IBD and CRC pathogenesis, such as circ_103516 (increased in IBD, AUC = 0.76), are novel biomarkers. EccDNA, with high immunostimulatory activity, is also increased in UC patients and DSS-induced colitis models, holding potential for monitoring disease severity and predicting treatment response [27, 28].

Minea et al. (2024) reviewed how genetic factors (NOD2, ATG16L1, IL-23R, CARD9, HLA) and epigenetic factors (DNA methylation, lncRNAs, miRNAs) contribute to IBD pathogenesis. LncRNAs like ANRIL (differentiates CD from controls with Sn 86.1% and Sp 64.2%) and miRNAs like miR-146b-5p (AUC = 0.869 for IBD) are promising for diagnosis and prognosis. Stemmer et al. (2024), using mega-analysis and machine learning, identified 34 genes distinguishing IBD from controls, including 3 novel lncRNAs (ENSG00000285744, ENSG00000287626, MIR4435−2HG) [29]. ML models achieved high accuracy (up to 0.947 for UC vs. control), with 12 genes showing significant upregulation in blood (P < 0.01) for severe endoscopic IBD. Stankovic et al. (2021) highlighted that ML models with omics data can predict IBD risk (AUC up to 0.86) and differentiate subtypes (AUC up to 0.97 for MMP9 in children) [30].

### Protein Biomarkers

Serum and fecal protein biomarkers are widely studied. Hashash et al. (2022) showed that an elevated serum globulin fraction (>4 g/dL) in 25.2% of IBD patients correlated significantly with severity biomarkers (CRP, ESR, anemia, hypoalbuminemia, all P < 0.001) and increased healthcare utilization [31]. In multivariate analyses, elevated globulin fraction was associated with hospitalization in CD (AOR 1.413, P = 0.031) and UC (AOR 1.799, P = 0.018), and surgery in CD (AOR 1.559, P = 0.006).

Ondriš et al. (2024) highlighted serum calprotectin (SC) and leucine-rich alpha-2 glycoprotein (LRG) as highly promising. SC correlated with clinical and endoscopic activity (AUROC 0.764 for relapse, Sn 72%, Sp 77%). LRG was superior to CRP in UC (LRG 0.73 vs. CRP 0.63 in UC) and effective in assessing endoscopic activity in small bowel CD [32]. The combination of OSM and FC achieved an AUC of 0.93 for IBD diagnosis (Cao et al., 2021). Wang et al. (2025) reinforced FC’s high diagnostic accuracy (Sn 91%, Sp 90%) for differentiating IBD from functional disorders, its correlation with endoscopic activity, and its ability to predict relapse (up to 96%) [33].

Vitali et al. (2023) identified Chymotrypsin C, Gelsolin, and RhoGDI2 as novel fecal biomarkers. Levels of these proteins significantly increased in IBD (P < 0.001) and correlated with endoscopic scores. Gelsolin (AUC 0.988, P < 0.0002) and RhoGDI2 (AUC 1.000, P < 0.0001) for CD, and RhoGDI2 (AUC P = 0.0004) for UC, had higher sensitivity and specificity than FC. Tobi et al. (2025) evaluated the innate immune system, showing that the FERAD ratio (serum ferritin/fecal p87) did not differentiate UC from CD but was significantly lower in IBD compared to controls (P < 0.00000071 for UC vs. controls; P < 3 × 10 ⁻ ⁶ for CD vs. controls), indicating innate immune system deficiency in IBD [20].

### Imaging Biomarkers

Bane et al. (2021) reviewed imaging biomarkers in CD, highlighting diffusion-weighted MRI (DWI) for intestinal inflammation (Sn 92.9%, Sp 91%). The apparent diffusion coefficient (ADC) correlated negatively with SES-CD (r = −0.76 to −0.88). Dynamic contrast-enhanced MRI (DCE-MRI) identified marked fibrosis (AUROC = 0.93) [34].

Furthermore, Teixeira et al. (2025) explored biosensors’ potential, highlighting portable devices that detect calprotectin in sweat (LOD of 0.1 µg/mL) and CRP in blood (LOD of 0.7 ng/mL), offering non-invasive, real-time solutions [35].

### Oxidative Stress

Tratenšek et al. (2024) conducted a meta-analysis revealing significant accumulation of oxidative damage biomarkers (MDA: SMD 1.20, P < 0.001) and reductions in antioxidants (albumin: SMD −1.20, P < 0.001) in active and inactive IBD versus controls. This suggests oxidative stress involvement in IBD pathogenesis [36].

### Predictive Performance for Therapeutic Response and Prognosis

• Plasma histidine levels in remission can predict the likelihood of relapse within 1 year [24].
• The gut microbiota can predict therapeutic response [19]. For example, *Bifidobacterium*, *Clostridium colinum*, *Eubacterium rectale* (VP+ of 1) were predictors of anti-TNF response.
• ML models identified 17 serum proteins to predict penetrating and stricturing complications in pediatric CD with an AUC of 0.88 [18].
• Fecal OSM and FC predicted non-response to infliximab with an AUC of 0.859 at week 28 [37].
• SC proved promising for predicting clinical remission (Sn 65.6%, Sp 67.6%) and mucosal healing (Sn 61.9%, Sp 80.9%) [32].

In summary, the analysis of the 27 articles reveals a vibrant landscape of emerging biomarkers, driven by advances in omics technologies and machine learning. These markers promise to transform IBD diagnosis and assessment, offering more precise, less invasive, and more predictive tools.

To facilitate interpretation of the evidence, Table 4 summarizes the main biomarker groups identified in this scoping review, together with their principal clinical applications and key limitations.

**Table 4:** Summary of the main emerging biomarker groups, clinical applications, and limitations in inflammatory bowel disease.

Biomarker group	Examples	Main use	Main limitation
Metabolomic	Nervonic acid, arachidonic acid, adrenic acid, histidine	Diagnosis, activity assessment, relapse prediction	Needs external validation and standardized assays
Microbiota	Akkermansia muciniphila, Faecalibacterium prausnitzii, Escherichia coli	Phenotyping, diagnosis, therapeutic response	High interindividual variability and sampling bias
Genetic/epigenetic	circRNAs, eccDNA, miRNAs, lncRNAs, NOD2-related markers	Risk stratification, prognosis	Limited clinical translation and population specificity
Protein	Calprotectin, LRG, OSM, gelsolin, RhoGDI2	Diagnosis, monitoring, mucosal healing	Heterogeneous thresholds and assay variability
Imaging/biosensors	DWI MRI, DCE-MRI, sweat calprotectin sensors	Non-invasive assessment	Limited validation and real-world availability
ML-based models	Gene/protein prediction panels	Classification and prediction	Overfitting risk and lack of multicenter validation

## 4. Discussion

The rising prevalence and chronic, complex nature of Inflammatory Bowel Diseases (IBD) have intensified the search for emerging biomarkers. These biomarkers aim to revolutionize the diagnosis and assessment of intestinal inflammatory lesions. This review, consolidating findings from 27 recent articles (2021–2025), reveals a promising and multifaceted panorama, with significant advances across several fronts, from metabolomics and microbiomics to genetic, protein, and imaging approaches. The results highlight a transition from non-specific markers to more precise, less invasive, and more predictive tools, supported by scientific evidence.

### 4.1. Advances in Metabolomics and Understanding of IBD Biochemical Profile

Metabolomics emerges as a powerful tool for elucidating altered biochemical profiles in IBD. Boye et al. (2024) emphasize that mass spectrometry (MS) and nuclear magnetic resonance (¹H NMR) offer crucial insights, identifying abnormal metabolic patterns [24]. Consistently observed are elevated amino acid levels and reduced short-chain fatty acids (SCFAs), corroborating systemic and local metabolic imbalance. Predictive models based on metabolomics demonstrated high accuracy (AUC > 0.8) in distinguishing IBD from non-IBD conditions and differentiating Ulcerative Colitis (UC) from Crohn’s Disease (CD). Notably, an amino acid panel, using LC-MS, achieved 88.4% sensitivity and 84.6% specificity in differentiating UC from CD [24]. Plasma histidine, in turn, proved to be a predictor of relapse in patients in remission, with an AUC of 0.83 in multivariate models [24].

Expanding this perspective, specific fecal fatty acids gain prominence. Kunst et al. (2024) identified fecal nervonic acid as a promising biomarker, with significantly higher levels in IBD patients (P < 0.001) and an AUROC of 0.827 for differentiating IBD from controls. This marker correlated positively with serum CRP (r = 0.376, P < 0.01) and FC (r = 0.575, P < 0.001), indicating its relationship with inflammation [22]. Huss et al. (2025) deepened this research, finding dihomo-γ-linolenic (DGLA), arachidonic (AA), and adrenic (AdA) acids elevated in the feces of IBD patients (AA: AUROC 0.859, P < 0.001; AdA: AUROC 0.841, P < 0.001) [23]. The correlation of these fatty acids with FC (AA: r = 0.507, P < 0.001; AdA: r = 0.480, P < 0.01) suggests a direct role in intestinal mucosal inflammation. Interestingly, the maintenance of normal levels of precursors of these fatty acids, such as linoleic acid, indicates that the dysfunction may reside more in downstream processing than in primary production [23].

Regarding lipoproteins, Zhao et al. (2025) demonstrated that Chinese IBD patients had significantly lower serum LDL and HDL levels compared to controls (P < 0.001 for both). HDL correlated negatively with disease activity (HBI in CD: r = −0.341, P < 0.001; Mayo clinical scores in UC: P < 0.001), and its specificity for predicting moderate-to-severe UC reached 87.34%, outperforming CRP. These findings indicate that the serum lipid profile reflects the inflammatory state and can assist in assessing IBD activity [25].

Tratenšek et al.‘s meta-analysis (2024) solidified oxidative stress’s role in IBD. It revealed significant accumulation of oxidative damage biomarkers (e.g., MDA, AOPP) and reduced antioxidants (e.g., PON-1, catalase, albumin, transferrin) in active and inactive IBD patients. MDA, for example, was 1.85 times higher in active IBD compared to controls. PON-1 showed a significant reduction in active CD (SMD −1.20) and UC (SMD −0.96), highlighting oxidative imbalance as a central marker in IBD pathogenesis [36].

### 4.2. The Gut Microbiota as a Diagnostic and Prognostic Pillar

The gut microbiota’s role in IBD is undeniable; it functions as both an etiological factor and a crucial biomarker. Wang et al. (2024) point out that dysbiosis characterizes IBD, and microbial profiles can predict postoperative recurrence and therapeutic response. *Akkermansia muciniphila* depletion in pediatric CD, and *Faecalibacterium prausnitzii* and *Escherichia coli* for phenotypic categorization of CD (Sn 82.50% for differentiating ileal from colonic CD), are notable examples [19]. Stemmer et al. (2024), through a mega-analysis, identified 34 genes differentiating IBD from controls, underscoring dysbiosis’s genetic Signature [38].

Zhao et al. (2024), using Mendelian randomization, established causal relationships between microbiota species and circulating inflammatory proteins in IBD risk. Genera such as *Candidatus Soleaferrea* and *Ruminococcaceae UCG013* were associated with a significant increase in IBD risk (OR = 1.227, P = 0.001 and OR = 1.293, P = 0.002, respectively), while *Clostridiaceae1* and *Ruminococcaceae UCG002* showed protective effects. Notably, IL-10Rα and MMP-10 mediate the effects of *Clostridiaceae1* in IBD, with a mediation proportion of 22.73% and 17.61%, respectively [21].

Sharma et al. (2025) and Wang et al. (2024) reiterate reduced microbial diversity and altered microbiota composition (decreased *F. prausnitzii*, increased *E. coli*) in IBD. This dysbiosis impacts SCFAs and bile acids metabolism, as well as the expression of morphogens, glycosylation, and podoplanin (PDPN), which in turn exacerbate inflammation and oxidative stress [19, 26].

### 4.3. Genetic and Epigenetic Biomarkers: New Frontiers

Genetics and epigenetics provide crucial insights into IBD heterogeneity and treatment individualization. Minea et al. (2024) highlight that SNPs, lncRNAs, and miRNAs are promising diagnostic and prognostic biomarkers. Risk alleles such as those of NOD2 (R702W, G908R, L1007fs) increase CD risk by 15-40x. DNA methylation, exemplified by THRAP2 and GBGT1, differentiates IBD patients from controls, while lncRNAs (e.g., ANRIL) and miRNAs (e.g., miR-21, miR-31, miR-155, miR-223) modulate inflammation and healing [29].

Xu et al. (2021) and Petito et al. (2024) introduce circular RNAs (circRNAs) and extrachromosomal circular DNA (eccDNA) as new biomarker classes [27, 28]. Circ_103516, for example, correlates with disease activity in IBD (AUC = 0.76), and its dysfunction links to colitis-associated colorectal cancer (CAC) pathogenesis. EccDNA, a type of circulating free DNA (cfDNA), possesses high immunostimulatory activity, inducing pro-inflammatory cytokines (IFN-1α, IFN-1β, IL-6, TNF-α), and is elevated in UC patients and colitis models [28]. EccDNA’s stability in blood makes it a promising prognostic biomarker, usable for monitoring disease severity and screening for CAC.

Machine learning (ML) has been instrumental in leveraging omics data. Stemmer et al. (2024) demonstrated that ML models identified 34 genes distinguishing inflamed IBD biopsies from controls with high accuracy (0.947 for UC vs. control), including three novel lncRNAs (ENSG00000285744, ENSG00000287626, MIR4435−2HG) [38]. Additionally, 12 genes showed significant upregulation in blood samples (P < 0.01) from patients with severe endoscopic IBD, suggesting their potential as non-invasive biomarkers [38]. Stankovic et al. (2021) reinforce that ML models with genomic and transcriptomic data exhibit robust predictive performance (AUC of 0.7 to 0.95), capable of identifying risk genes, classifying IBD subtypes, and predicting treatment response [30].

### 4.4. Protein Biomarkers: From Serum to Feces

Protein biomarkers continue to be an area of intense research, focusing on their specificity and ability to reflect local inflammation. Calprotectin, both fecal (FC) and serum (SC), remains a central focus. Jukic et al. (2021) and Wang et al. (2025) highlight FC as a widely used marker for intestinal inflammation (Sn 92.9%, Sp 91%), effective in differentiating IBD from non-inflammatory diseases (e.g., IBS) [33, 39]. FC levels < 40 µg/g effectively exclude IBD, while values between 150–250 µg/g represent a “gray zone” [39]. Ondriš et al. (2024) and Cao et al. (2021) show that SC and LRG are promising, with SC correlating with clinical and endoscopic activity (AUROC 0.85, Sn 83.3%, Sp 81.25% for active UC) and LRG outperforming CRP in assessing UC endoscopic activity [32, 37]. Cao et al. (2021) observed that combining OSM and fecal FC improves IBD diagnosis (AUC = 0.93) and identifies mucosal healing (AUC = 0.923) [37].

Vitali et al. (2023) identified Chymotrypsin C, Gelsolin, and RhoGDI2 as novel fecal biomarkers. Levels of these proteins significantly increased in IBD (P < 0.001) and correlated with endoscopic scores [20]. Gelsolin (AUC 0.988, P < 0.0002) and RhoGDI2 (AUC 1.000, P < 0.0001) for CD, and RhoGDI2 (AUC P = 0.0004) for UC, had higher sensitivity and specificity than FC. Tobi et al. (2025) evaluated the innate immune system, showing that the FERAD ratio (serum ferritin/fecal p87) did not differentiate UC from CD but was significantly lower in IBD compared to controls (P < 0.00000071 for UC vs. controls; P < 3 × 10 ⁻ ⁶ for CD vs. controls), indicating innate immune system deficiency in IBD [40].

Ungaro et al. (2021) used machine learning to identify 17 serum proteins predicting penetrating and stricturing complications in pediatric CD, with an AUC of 0.88. Relevant proteins include CCL17, GDF15, IL1RA, MMP9, and VEGFA, providing targets for early interventions [18]. Nowak et al. (2023) also reviewed PR3-ANCA (Sn 75%, Sp 69% for active UC) and anti-integrin αvβ6 autoantibodies (Sn 76.3%, Sp 96% for UC vs. IBS), which show promise for UC diagnosis and can even predict disease development up to 10 years before clinical onset [41].

### 4.5. Imaging Biomarkers

MRI remains a cornerstone in IBD assessment. Bane et al. (2021) extensively reviewed quantitative MRI biomarkers for CD. Diffusion-weighted MRI (DWI) achieved sensitivity of 92.9% and specificity of 91% for intestinal inflammation [34]. The apparent diffusion coefficient (ADC) correlated negatively with endoscopic activity scores such as SES-CD (r = −0.76 to −0.88). Dynamic contrast-enhanced MRI (DCE-MRI) assessed tissue vascularization, and magnetization transfer MRI (MT-MRI) detected fibrosis. The Magnetization Transfer Ratio (MTR) strongly correlated with histological fibrosis (r = 0.77, P < 0.0001) and discriminated mild/moderate from moderate/severe fibrosis with an AUROC of 0.92 [34].

Teixeira et al. (2025) highlight biosensors’ potential for non-invasive, real-time monitoring. Portable devices can detect calprotectin in sweat (LOD of 0.1 µg/mL) and CRP in blood (LOD of 0.7 ng/mL). Headspace gas sensors differentiate IBS from IBD with sensitivity 76% and specificity 88% [35].

### 4.6. Implications for diagnosis and assessment of intestinal inflammatory lesions

In summary, research on emerging IBD biomarkers points to an era of more precise diagnosis and individualized assessment. The combination of metabolomic, microbiological, genetic, epigenetic, protein, and imaging approaches offers a holistic view of IBD pathogenesis. The high sensitivity and specificity of many new biomarkers, coupled with their non-invasive nature, promise to reduce reliance on endoscopic procedures and significantly improve patient quality of life. The ability to predict disease activity, prognosis, complication risk, and therapeutic response will allow for earlier, more personalized interventions, aligning with precision medicine principles.

However, despite promising results, many biomarkers still require validation in large independent cohorts and standardization of methods for clinical implementation. IBD’s heterogeneity and complex interactions demand a multifactorial approach, where integrating different biomarker types can provide the most complete and effective model for the future.

Although the biomarker landscape is promising, clinical translation remains limited by heterogeneity in study design, populations, biospecimens, analytical platforms, and outcome definitions. This variability reduces comparability across studies and makes external validation essential before routine clinical use.

Machine learning-based approaches show encouraging performance, but most studies are exploratory, single-cohort, and vulnerable to overfitting. Their clinical value depends on multicenter validation, transparent feature selection, and reproducible calibration in independent populations.

### 4.7. Limitations and Future Perspectives

Despite notable advances in IBD biomarker research, the reviewed studies reveal important limitations that shape future perspectives. Heterogeneity of populations, methodological designs, and analytical variability persist as significant challenges [18, 21]. Many promising markers still lack absolute specificity for IBD compared to other inflammatory conditions, such as fecal calprotectin (FC) and oncostatin M (OSM) [33, 39, 42]. Validation in large prospective and independent cohorts, as well as standardization of cut-offs and detection methods, are crucial steps often missing for widespread clinical implementation [32, 36]. Furthermore, the complexity and cost of some omics technologies and advanced imaging [34, 35] represent barriers to large-scale adoption. Distinguishing UC and CD, influenced by tissue factors, also remains an obstacle [38].

Future perspectives are optimistic and align with precision medicine principles. The integration of multi-omics approaches (metabolomics, microbiomics, genomics, transcriptomics, proteomics) with detailed clinical data is seen as the path to patient stratification and personalized interventions [24, 29, 38]. Machine Learning (ML) will be fundamental for processing and interpreting these complex datasets, identifying molecular signatures and outcome predictors [18, 30]. The focus on non-invasive detection and continuous monitoring, using fecal [20, 22, 23], serum [25, 32], and biosensor [35] biomarkers, aims to reduce the need for invasive procedures. Innovative biomarkers such as circRNAs [27] and eccDNA [28] promise new diagnostic and therapeutic avenues. Ultimately, international collaboration and research standardization are essential to translate these promising findings into tangible benefits for IBD patients.

## Conclusion

This review, consolidating the findings of 27 recent articles, demonstrates a substantial transformation in the landscape of biomarkers for Inflammatory Bowel Disease (IBD). Current research transcends traditional inflammatory markers, revealing a complex ecosystem of biomarkers with superior potential for diagnosis, disease activity monitoring, and prediction of clinical outcomes.

In the metabolomic domain, the identification of fecal fatty acids, such as nervonic and arachidonic acid, and changes in serum lipid profiles, including LDL and HDL, offer new lenses to assess the inflammatory state and the risk of disease progression. These analyses demonstrate remarkable discriminatory capacity and correlation with IBD activity.

The gut microbiota emerges as a central pillar, with dysbiosis signatures and specific microbial profiles identified as crucial biomarkers for differentiating IBD subtypes, predicting recurrence, and therapeutic response. The use of advanced approaches, such as Mendelian randomization and machine learning, has allowed the establishment of causal relationships and the identification of genetic and protein panels directly influenced by microbial composition, paving the way for precision medicine.

Genetic and epigenetic biomarkers, including lncRNAs and eccDNA, are providing in-depth insights into pathogenic mechanisms and the risk of progression. The integration of omics data through machine learning has enabled the identification of genes and pathways that distinguish IBD patients from controls with high accuracy, as well as the stratification of disease profiles.

Protein biomarkers, both serum and fecal, continue to evolve. Serum globulin fractions and novel fecal markers such as Chymotrypsin C, Gelsolin, and RhoGDI2 have demonstrated remarkable sensitivity and specificity. In some contexts, they surpass conventional biomarkers in identifying and monitoring intestinal inflammation. The evaluation of circulating inflammatory proteins and validation of new protein panels are fundamental to refine the prediction of clinical outcomes and response to specific therapies.

In sum, the convergence of diverse biomarker platforms, coupled with sophisticated analytical tools such as artificial intelligence, is paving the way for a more holistic understanding and more precise management of IBD. Although rigorous validation in large cohorts and standardization of methodologies remain imperative, recent advances solidify the promise of an era where early diagnosis, personalized assessment, and non-invasive monitoring will become the backbone of IBD patient care.

### Declaration of AI and AI-assisted technologies in the writing process

The authors declare that no generative artificial intelligence (AI) or AI-assisted technologies were used in the writing process of this manuscript.

## Supporting information

S1 File Supplementary material for this article includes 28 detailed tables containing comprehensive data extraction from each included study. These tables provide granular information on study characteristics, biomarker details, and quantitative results, serving as a rich resource for readers.
(DOCX)

S2 File PRISMA Checklist. (DOCX)
