# Supplementary material for: A scoping review on emerging biomarkers in inflammatory bowel disease: Towards precision medicine in diagnosis and therapeutic management
Source: PLoS One. 2026 Jul 13;21(7):e0353295. doi: 10.1371/journal.pone.0353295 (PMC13362126; doi:10.1371/journal.pone.0353295)
Supplement: S1 File — These tables provide granular information on study characteristics, biomarker details, and quantitative results, serving as a rich resource for readers. (DOCX) [file pone.0353295.s001.docx]

**SUPPLEMENTARY MATERIAL**

**Detailed Data of Selected Articles – Data extracted by the DATA EXTRACTION INSTRUMENT – Table 3 (Manuscript)**

**Table 1. Article 1 Metabolomics for enhanced clinical understanding of inflammatory bowel disease**

| Category | Variable | Description |
| --- | --- | --- |
| Study Identification | Lead Author | Boye, T.L. |
|  | Year of Publication | 2024 |
|  | Country | Denmark, Singapore |
|  | Journal | Life Sciences |
|  | Study Type | Comprehensive Review |
|  | Level of Evidence | Not directly applicable, as it is a review. Consolidates evidence from various primary studies. |
| Population | Sample Size | Not applicable (review of primary studies with varied populations) |
|  | Age (mean ± SD) | Not applicable (variable among reviewed studies) |
|  | Sex (%) | Not applicable (variable among reviewed studies) |
|  | IBD Type | Crohn’s Disease (CD), Ulcerative Colitis (UC), unclassified IBD (IBD-U) |
| Biomarkers | Biomarker Type | Low molecular weight metabolites (amino acids, lipids/lipoproteins, metabolic intermediates, small proteins) |
|  | Biomarker Name | Mass Spectrometry (MS), Proton Nuclear Magnetic Resonance (1H NMR) spectroscopy, Short-chain fatty acids (SCFAs), Amino acids (e.g., Histidine), Lysophosphatidylcholines (LPC), Phosphatidylcholines (PC), Bile acids. |
|  | Detection Method | Mass Spectrometry (MS), Proton Nuclear Magnetic Resonance (1H NMR) |
|  | Sample Type | Plasma, serum, urine, feces, tissue biopsies. |
|  | Diagnostic Purpose | Diagnosis, prognosis, monitoring of disease activity, treatment response, prevention, identification of new biomarkers. |
| Results | Sensitivity (%) | Diagnosis: Predictive models differentiate IBD from non-IBD and IBD subtypes with AUC > 0.8. Targeted amino acid analysis using LC-MS resulted in a predictive panel to distinguish UC and CD with 88.4% sensitivity. |
|  | Specificity (%) | Diagnosis: The same panel resulted in 84.6% specificity. |
|  | Positive Predictive Value (%) | Prognosis: - Plasma histidine levels in remission can predict the likelihood of relapse within 1 year. - A combined metabolomics and proteomics model predicted relapse in 2 years with AUC of 0.83. Treatment Response: - Metabolic profiles predict response to anti-TNF therapy: - Fecal lipids: AUC = 0.94. - Serum lipids: AUC = 0.78. - Bile acids: AUC = 0.81. |
|  | Negative Predictive Value (%) | Not directly specified. |
| Methodological Quality | Control Group | Primary studies included healthy controls. |
|  | Blinding | Not directly applicable for review. |
|  | Reported Limitations | Current technologies do not capture the entire metabolome. Metabolite identification depends on databases. Variability and low sensitivity for certain metabolites. Difficulty in structural elucidation in untargeted analyses. Low sensitivity of NMR compared to LC-MS. Variable reproducibility in large-scale studies. Need for large cohorts and validation for consistent biomarkers. |

**Table 2. Article 2 - The emerging role of the gut microbiota and its application in inflammatory bowel disease**

| Category | Variable | Description |
| --- | --- | --- |
| Study Identification | Lead Author | Wang, X. |
|  | Year of Publication | 2024 |
|  | Country | China |
|  | Journal | Biomedicine & Pharmacotherapy |
|  | Study Type | Comprehensive Review |
|  | Level of Evidence | Not directly applicable for review. Consolidates evidence from various primary studies. |
| Population | Sample Size | Not applicable (review of primary studies with varied populations) |
|  | Age (mean ± SD) | Not applicable (variable among reviewed studies) |
|  | Sex (%) | Not applicable (variable among reviewed studies) |
|  | IBD Type | Crohn’s Disease (CD), Ulcerative Colitis (UC) |
| Biomarkers | Biomarker Type | Microorganisms (bacteria, viruses, fungi) and their metabolites |
|  | Biomarker Name | Akkermansia muciniphila, Faecalibacterium prausnitzii, Escherichia coli, Fusobacteria, Proteus, Oscillibacter, Ruminiclostridium 6, Salmonella phage SEN4, Streptococcus phage YMC-2011, Enterococcus, Lactobacillus, Eggerthella, Clostridium cluster XVIII, Thomasclavelia ramosum, Thomasclavelia spiroforme, Thomasclavelia saccharogumia, Firmicutes CAG 83, cucumber green mottle mosaic virus. |
|  | Detection Method | qPCR, 16S rRNA sequencing, metagenomic shotgun sequencing, whole-metagenome sequencing, 2D-PCR, multi-omics profiling. |
|  | Sample Type | Feces, mucosal biopsies (ileal, colonic), colonic lavage fluid, serum, mucosal brushings, saliva. |
|  | Diagnostic Purpose | Diagnosis, prognosis, prediction of postoperative recurrence, prediction of therapeutic response. |
| Results | Sensitivity (%) | A. muciniphila: May be a biomarker for early-onset pediatric CD. F. prausnitzii: In ileal CD, AUC > 0.772, Sn 82.50% to differentiate from colonic CD. E. coli: In CD, Sn 82.50% to differentiate colonic CD from extensive UC. F. prausnitzii: In CD vs. controls, Sn 62%. F. prausnitzii phylogroup I: In CD vs. controls, Sn 91.48%. Bacteroides species (SNVs): In CD, AUC 0.77. Christensenellaceae and Enterobacteriaceae: In pediatric UC, AUC 0.869 (16S genus), AUC 0.763 (shotgun species). Phage (Salmonella phage SEN4, Streptococcus phage YMC-2011, crAssphage): In UC, AUC 94.5%. Bacteria (all detected): In UC, AUC 97.6%. Clostridium cluster XVIII, Thomasclavelia ramosum, T. spiroforme, T. saccharogumia: In IBD, Sn 0.769. Combined omics: In IBD, AUC 0.80 (95% CI: 0.63, 0.98), Nagelkere R² 0.46. |
|  | Specificity (%) | F. prausnitzii: In ileal CD, Sp 57.14% to differentiate from colonic CD. E. coli: In CD, Sp 53.84% to differentiate colonic CD from extensive UC. F. prausnitzii: In CD vs. controls, Sp 80%. F. prausnitzii phylogroup I: In CD vs. controls, Sp 73.02%. Bacteria (all detected): In UC, Sp 97.6%. Clostridium cluster XVIII, Thomasclavelia ramosum, T. spiroforme, T. saccharogumia: In IBD, Sp 0.853. Fungi and bacteria + demographic data: In IBD, AUC 0.842. |
|  | Positive Predictive Value (%) | Bifidobacterium, Clostridium colinum, Eubacterium rectale: In anti-TNF response, PPV of 1. Vibrio, uncultured Clostridiales: In anti-TNF response, PPV of 0.8. Fusobacteria: Primary cause of early POR (Postoperative Recurrence). AIEC: Predicted severe (aOR [2.54 (95% CI 1.01–6.44), p=0.049]) and very severe (aOR [3.36 (95% CI 1.25–9.06), p=0.017]) endoscopic POR. Oscillibacter and Ruminiclostridium 6: Elevated levels correlated with pro-inflammatory cytokines (IL-6, IL-1β, TNF-α). |
|  | Negative Predictive Value (%) | Streptococcus mitis, Vibrio, uncultured Clostridiales: In anti-TNF response, NPV of 0.8. Bifidobacterium, Clostridium colinum, Eubacterium rectale: In anti-TNF response, NPV of 1. |
| Methodological Quality | Control Group | Healthy controls were included in all reviewed primary studies. |
|  | Blinding | Not directly applicable for review. |
|  | Reported Limitations | Fecal sampling does not reflect the entire gut microbiota. Interindividual variations. Small sample sizes in many studies. Lack of control over environmental variables. Absence of longitudinal data. Inconsistency of results in standard treatments. Challenges in standardizing protocols for microbiota isolation/quantification/data analysis. High cost of omics technologies. |

**Table 3. Article 3 - Mendelian randomization reveals predictive, preventive, and personalized insights into inflammatory bowel disease: the role of gut microbiome and circulating inflammatory proteins**

| Category | Variable | Description |
| --- | --- | --- |
| Study Identification | Lead Author | Zhao, W. |
|  | Year of Publication | 2024 |
|  | Country | China |
|  | Journal | EPMA Journal |
|  | Study Type | Bidirectional and multivariate Mendelian Randomization (MR) study (MVMR) |
|  | Level of Evidence | Mendelian Randomization (high level of evidence for causal inference) |
| Population | Sample Size | IBD: 9083 cases, 403098 controls (total 412181); UC: 9083 cases, 403098 controls (total 412181); CD: 2033 cases, 409940 controls (total 411973). All of European ancestry (FinnGen Biobank). |
|  | Age (mean ± SD) | Not specified in the provided study data. |
|  | Sex (%) | Not specified in the provided study data. |
|  | IBD Type | Crohn’s Disease (CD), Ulcerative Colitis (UC) and general IBD. |
| Biomarkers | Biomarker Type | Gut microbiota (genus, family, class, phylum) and circulating inflammatory proteins. |
|  | Biomarker Name | Microbiota: Genus Actinomyces, Genus Candidatus Soleaferrea, Phylum Bacteroidetes, Genus Ruminococcaceae UCG013, Class Clostridia, Family Clostridiaceae1, Phylum Cyanobacteria, Genus Ruminococcaceae UCG002, Class Lentisphaeria, Genus Turicibacter, Order Victivallales, Family Peptococcaceae, Family Rikenellaceae, Genus Anaerostipes, Family Bacteroidaceae, Genera Bacteroides, Eisenbergiella, Phascolarctobacterium, Ruminiclostridium 5, Ruminococcus torques, Subdoligranulum, Order Bacillales, Family Lachnospiraceae, Genus Adlercreutzia, Phylum Euryarchaeota, Phylum Verrucomicrobia, Genus Olsenella, Genus Lachnospiraceae UCG010, Genus Desulfovibrio, Genus Eubacterium nodatum group, Genus Haemophilus, Genus Lachnospiraceae UCG004, Genus Lachnospiraceae NK4A136 group. Proteins: Interleukin-10 receptor subunit alpha (IL-10Rα), Matrix metalloproteinase-10 (MMP-10), Leukemia inhibitory factor (LIF), Tumor necrosis factor superfamily ligand-12 (TNFSF12), Interleukin-17C (IL-17C), TNF-related activation-induced cytokine (TRANCE), Axin-1 (AXIN1), T-cell surface glycoprotein CD5 (CD5), CCL19, Interleukin-12 subunit beta (IL12B), CD6, CX3CL1, CXCL10. |
|  | Detection Method | Mendelian Randomization (MR) and Multivariate Mendelian Randomization (MVMR) using GWAS (Genome-Wide Association Study) data. |
|  | Sample Type | Genetic data (SNPs) associated with gut microbiota and circulating inflammatory proteins. |
|  | Diagnostic Purpose | Identification of causal factors (microbiota and inflammatory proteins) for IBD, UC, and CD risk. Verification of mediation pathways for disease development. |
| Results | Sensitivity (%) | Not directly applicable in MR for classic diagnostic test sensitivity and specificity, but rather in strength of causal association. |
|  | Specificity (%) | Not directly applicable. |
|  | Positive Predictive Value (%) | IBD: Risk factors (OR > 1): Genus Candidatus Soleaferrea (OR = 1.227, 95% CI = 1.090–1.381, P = 0.001), Genus Ruminococcaceae UCG013 (OR = 1.293, 95% CI = 1.098–1.521, P = 0.002). Risk proteins (OR > 1): AXIN1, CD5, CCL19, IL-10Rα, MMP-10, TNFSF12. Mediation: IL-10Rα (22.73% mediation) and MMP-10 (17.61% mediation) mediate the effect of Family Clostridiaceae1 on IBD. UC: Risk proteins (OR > 1): IL-10Rα, TNFSF12. Mediation: IL-10Rα mediates the effect of Family Clostridiaceae1 on UC (15.91% mediation). CD: Risk factors (OR > 1): Genus Ruminococcaceae UCG013 (OR = 1.955, 95% CI = 1.391–2.749, P < 0.001). |
|  | Negative Predictive Value (%) | Not directly applicable in MR. |
| Methodological Quality | Control Group | Healthy individuals from FinnGen Biobank. |
|  | Blinding | MR inherently acts as a "natural randomization" due to the random allocation of genetic variants at birth, mitigating confounders. |
|  | Reported Limitations | Genetic data primarily from European (Finnish) populations, limiting generalization to other ethnicities. Relatively small sample size for UC and CD subtypes. Some inflammatory proteins (e.g., LIF, TNF-RAIL) did not maintain significance after multivariate adjustments. |

**Table 4. Article 4 - CircRNAs as promising biomarkers of inflammatory bowel disease and its associated-colorectal cancer**

| Category | Variable | Description |
| --- | --- | --- |
| Study Identification | Lead Author | Xu, Y. |
|  | Year of Publication | 2021 |
|  | Country | China, Ghana |
|  | Journal | Am J Transl Res |
|  | Study Type | Comprehensive Review |
|  | Level of Evidence | Not directly applicable for review. Consolidates evidence from various primary studies. |
| Population | Sample Size | Not applicable (review of primary studies with varied populations) |
|  | Age (mean ± SD) | Not applicable (variable among reviewed studies) |
|  | Sex (%) | Not applicable (variable among reviewed studies) |
|  | IBD Type | Crohn’s Disease (CD), Ulcerative Colitis (UC), Colitis-Associated Colorectal Cancer (CAC) |
| Biomarkers | Biomarker Type | Circular RNAs (circRNAs) |
|  | Biomarker Name | circ_103516, circ_0007919, circ_004662, CDKN2B-AS1, circ_102610, circPACRGL, circ_0000069, circ_0020397, circ_001569, circ_0004585, circ_0001178, circ_0000826, circDDX17, circ_103809, circ_104700, circBANP, CCDC66, circ_102958. |
|  | Detection Method | Microarray analysis, bioinformatics, high-throughput sequencing. |
|  | Sample Type | PBMCs (peripheral blood mononuclear cells), colonic mucosal biopsies, serum. |
|  | Diagnostic Purpose | Diagnosis, prognosis, differentiation between IBD subtypes, prediction of CRC metastasis. |
| Results | Sensitivity (%) | circ_103516 (IBD): AUC 0.76. circ_004662 (CD/UC Differentiation): Potential candidate, Sn/Sp not specified. circPACRGL (CRC): Sn/Sp not specified. circ_0000069, circ_0020397, circ_001569 (CRC): Potential biomarkers, Sn/Sp not specified. circ_0004585 (CRC): Potential biomarker, Sn/Sp not specified. circ_0001178, circ_0000826 (Hepatic metastasis of CRC): Potential biomarkers, Sn/Sp not specified. |
|  | Specificity (%) | Not directly specified. |
|  | Positive Predictive Value (%) | circ_103516 (IBD): Positively correlated with disease activity (CDAI, Mayo, ESR) and inflammatory cytokines (TNF-α, IFN-γ). Negatively correlated with IL-10. circ_0007919 (UC): Decrease correlated with EPC1 and VIPR1, involved in mucosal inflammation. CDKN2B-AS1 (IBD): Decreased in IBD, improves barrier function by interacting with Claudin-2. circ_102610 (CD): Increased, promotes proliferation and EMT of epithelial cells. circPACRGL (CRC): Increased, promotes proliferation, migration, invasion, N1/N2 granulocytosis differentiation. circ_0000069, circ_0020397, circ_001569 (CRC): Increased, promote proliferation, inhibit apoptosis, increase invasiveness. circ_0004585 (CRC): Increased, positively correlated with tumor size. |
|  | Negative Predictive Value (%) | circDDX17 (CRC): Decreased, tumor suppressor. circ_103809, circ_104700 (CRC): Decreased, correlated with metastasis, differentiation, and perineural infiltration. circBANP (CRC): Increased, prognostic and therapeutic marker. CCDC66 (CRC): Increased, promotes growth and metastasis, negatively correlated with prognosis. circ_102958 (CRC): Increased, promotes proliferation, migration, invasion, prognostic marker. |
| Methodological Quality | Control Group | Healthy controls were included in the reviewed primary studies. |
|  | Blinding | Not directly applicable for review. |
|  | Reported Limitations | The exact mechanisms of circRNAs in IBD and CRC are not yet fully explored. The potential of circRNAs as biomarkers and therapeutic targets is still in the early stages of investigation. Detection can be expensive and complex. |

**Table 5. Article 5 - Elevated serum globulin fraction as a biomarker of multiyear disease severity in inflammatory bowel disease**

| Category | Variable | Description |
| --- | --- | --- |
| Study Identification | Lead Author | Hashash, J.G. |
|  | Year of Publication | 2022 |
|  | Country | USA, Lebanon, Greece |
|  | Journal | Annals of Gastroenterology |
|  | Study Type | Retrospective Observational Cohort Study (Prospective IBD registry) |
|  | Level of Evidence | Level II-2 (Cohort study, no randomization, externally validated results) |
| Population | Sample Size | 1767 IBD patients (446 with elevated globulin fraction, 1321 with normal globulin fraction) with 4 years of follow-up. |
|  | Age (mean ± SD) | 48.4 ± 15.1 years (mean for the total cohort) |
|  | Sex (%) | 53.5% female in the total cohort (59.4% female in the elevated globulin group vs. 51.5% in the normal globulin group, P = 0.004) |
|  | IBD Type | 65.4% Crohn’s Disease (CD), 34.5% Ulcerative Colitis (UC) |
| Biomarkers | Biomarker Type | Serum globulin fraction. |
|  | Biomarker Name | Globulin fraction (>4 g/dL), albumin, C-reactive protein (CRP), erythrocyte sedimentation rate (ESR), hemoglobin. |
|  | Detection Method | Routine clinical chemistry (subtraction of albumin from total protein). |
|  | Sample Type | Serum. |
|  | Diagnostic Purpose | Biomarker for disease severity and healthcare utilization. |
| Results | Sensitivity (%) | Not directly specified. |
|  | Specificity (%) | Not directly specified. |
|  | Positive Predictive Value (%) | Significant correlations (P < 0.001): - Median CRP: 1.4 (2.9) mg/L (elevated) vs 0.5 (0.7) mg/L (normal). - Median ESR: 27.3 (31.8) mm/h (elevated) vs 13.2 (17.3) mm/h (normal). - Anemia: 39.2% (elevated) vs 16.7% (normal). - Hypoalbuminemia: 76.5% (elevated) vs 35.7% (normal). Medication Use: - Biologics (UC): 36.2% (elevated) vs 20.1% (normal), P < 0.001. - Biologics (CD): 58.1% (elevated) vs 46.3% (normal), P < 0.001. Healthcare Utilization: - Emergency visits (UC): 63% (elevated) vs 33.5% (normal), P < 0.001. - Hospitalization (UC): 47.8% (elevated) vs 28.5% (normal), P < 0.001. - IBD-related surgery (UC): 18.8% (elevated) vs 10.4% (normal), P = 0.012. - Emergency visits (CD): 64.9% (elevated) vs 42.0% (normal), P < 0.001. - Hospitalization (CD): 55.8% (elevated) vs 36.7% (normal), P < 0.001. - IBD-related surgery (CD): 22.1% (elevated) vs 14.6% (normal), P = 0.003. Multivariate analysis (AOR - adjusted odds ratio): - Elevated globulin fraction and hospitalization (CD): AOR 1.413 (95% CI 1.033–1.934), P = 0.031. - Elevated globulin fraction and hospitalization (UC): AOR 1.799 (95% CI 1.104–2.931), P = 0.018. - Elevated globulin fraction and surgery (CD): AOR 1.559 (95% CI 1.334–2.134), P = 0.006. - Elevated globulin fraction and surgery (UC): AOR 1.229 (95% CI 0.683–2.211), P = 0.492 (not significant). Kaplan-Meier analysis: - Hospitalizations (CD): P < 0.001. - Hospitalizations (UC): P < 0.001. - IBD-related surgeries (CD): P < 0.001. - IBD-related surgeries (UC): P = 0.013. |
|  | Negative Predictive Value (%) | Not directly specified. |
| Methodological Quality | Control Group | Patients with normal globulin fraction (1321 patients). |
|  | Blinding | Not specified, but retrospective registry study, which may imply absence of blinding. |
|  | Reported Limitations | Observational study in a tertiary referral center (selection bias). Inclusion of patients at different disease stages. Immunological mechanisms associated with elevated globulin fraction were not fully characterized. Lack of universal standardization of disease severity measurement strategy. |

**Table 6. Article 6 - Precision Medicine in Inflammatory Bowel Disease: A Spotlight on Emerging Molecular Biomarkers**

| Category | Variable | Description |
| --- | --- | --- |
| Study Identification | Lead Author | Mestrovic, A. |
|  | Year of Publication | 2024 |
|  | Country | Croatia |
|  | Journal | Biomedicines |
|  | Study Type | Comprehensive Review |
|  | Level of Evidence | Not directly applicable for review. Consolidates evidence from various primary studies. |
| Population | Sample Size | Not applicable (review of primary studies with varied populations) |
|  | Age (mean ± SD) | Not applicable (variable among reviewed studies) |
|  | Sex (%) | Not applicable (variable among reviewed studies) |
|  | IBD Type | Crohn’s Disease (CD), Ulcerative Colitis (UC) |
| Biomarkers | Biomarker Type | Serum, serological, fecal, genetic, epigenetic, proteomic, metabolomic. |
|  | Biomarker Name | C-reactive Protein (CRP), Erythrocyte Sedimentation Rate (ESR), Leucine-Rich Alpha-2 Glycoprotein (LRG), pANCA, ASCA, Fecal Calprotectin (FC), Lactoferrin, S100A12, αvβ6 antibody, Prostaglandin E-Major Urinary Metabolite (PGE-MUM), microRNAs (miRNA-675-5p, miR-16-2-3p, miR-150-5p, miR-224-5p, miR-32-5p), Oncostatin M (OSM), B-cell Activating Factor (BAFF), Albumin, Fibrinogen, Serum Amyloid A (SAA), Globulin, α1-Acid Glycoprotein (AGP), TFF3, CPa9-HNE. |
|  | Detection Method | Immunoturbidimetry, spectrometry, ELISA, qPCR, multi-omics. |
|  | Sample Type | Serum, feces, urine. |
|  | Diagnostic Purpose | Diagnosis, assessment of disease activity, prediction of mucosal healing, prediction of therapeutic response, prediction of recurrence. |
| Results | Sensitivity (%) | αvβ6 antibody: - UC diagnosis: 92%; in UC vs. CD: 76.3%; in UC vs. IBS: 76.3%; in preclinical phases: AUROC of 0.8. miRNA-675-5p: - UC vs. HC: 85.7%; CD vs. HC: 88.4%. fecal BAFF: - IBD vs. HC and IBS: 90% (cut-off 325 pg/mL). - Distinguish IBS from IBD: 84%. S100A12 (pediatric): IBD diagnosis: 95%. Fecal calprotectin: IBD diagnosis (adults and children): 0.89. |
|  | Specificity (%) | αvβ6 antibody: - UC diagnosis: 94.8%; in UC vs. CD: 79%; in UC vs. IBS: 96%; in preclinical phases: AUROC of 0.8. miRNA-675-5p: - UC vs. HC: 97.3%; CD vs. HC: 95.2%. fecal BAFF: - IBD vs. HC and IBS: 96% (cut-off 325 pg/mL). - Distinguish IBS from IBD: 100%. S100A12 (pediatric): IBD diagnosis: 97%. Fecal calprotectin: IBD diagnosis (adults and children): 0.81. pANCA (distinguish UC from CD): 91%. |
|  | Positive Predictive Value (%) | PGE-MUM: - Prediction of endoscopic activity: Sn 81% (cut-off 21.8 µg/g·Cr). - Prediction of histological activity: Sn 82% (cut-off 17.0 µg/g·Cr). - Prediction of relapse: AUROC 0.721 (cut-off 25.2 mg/g Cr). miRNAs (predict glucocorticoid resistance): - miR-16-2-3p, miR-150-5p, miR-224-5p: Sp 97.3%. - miR-32-5p: Sn 97.4%. Oncostatin M (OSM): - Correlated with endoscopic and clinical activity. - Fecal OSM + FC: IBD diagnosis (AUROC 0.93), prediction of therapeutic response (AUROC 0.859). - Elevated serum OSM levels associated with anti-TNF therapy failure. |
|  | Negative Predictive Value (%) | FC: Normal levels (≤40 µg/g) have high NPV for IBD. |
| Methodological Quality | Control Group | Healthy controls and patients with other conditions (e.g., IBS, control disease) included in the reviewed primary studies. |
|  | Blinding | Not directly applicable for review. |
|  | Reported Limitations | Many markers are non-specific for IBD. High variability of S100A12 in adults. Lactoferrin studies with low sensitivity. Lack of validation for many new markers in large cohorts. Non-specificity of serum OSM. Lack of standardization of protocols and cut-offs. |

**Table 7. Article 7 - Fecal Nervonic Acid as a Biomarker for Diagnosing and Monitoring Inflammatory Bowel Disease**

| Category | Variable | Description |
| --- | --- | --- |
| Study Identification | Lead Author | Kunst, C. |
|  | Year of Publication | 2024 |
|  | Country | Germany |
|  | Journal | Biomedicines |
|  | Study Type | Cross-Sectional Observational Study (Clinical) |
|  | Level of Evidence | Level II-2 (Cohort study, no randomization) |
| Population | Sample Size | 62 IBD patients (38 Crohn’s Disease, 24 Ulcerative Colitis) and 17 healthy controls. |
|  | Age (mean ± SD) | IBD: 42 years (range 19-78); Controls: 48 years (range 23-78). No significant difference between groups. |
|  | Sex (%) | IBD: 28/34 (female/male); Controls: 10/7 (female/male). No significant difference between groups. |
|  | IBD Type | Crohn’s Disease (CD), Ulcerative Colitis (UC) |
| Biomarkers | Biomarker Type | Very long-chain fatty acids (VLCFAs) |
|  | Biomarker Name | Nervonic Acid (NA), Lignoceric Acid, Pentacosanoic Acid. |
|  | Detection Method | Gas Chromatography coupled with Mass Spectrometry (GC-MS). |
|  | Sample Type | Feces. |
|  | Diagnostic Purpose | Diagnosis of IBD, monitoring of disease activity. |
| Results | Sensitivity (%) | NA to differentiate IBD from controls: 71% (cut-off 0.49 µmol/g). NA to differentiate FC < 120 µg/g from FC ≥ 120 µg/g: 78% (cut-off 0.94 µmol/g). |
|  | Specificity (%) | NA to differentiate IBD from controls: 82% (cut-off 0.49 µmol/g). NA to differentiate FC < 120 µg/g from FC ≥ 120 µg/g: 82% (cut-off 0.94 µmol/g). |
|  | Positive Predictive Value (%) | Fecal NA levels in IBD patients: Significantly higher than in healthy controls (P < 0.001). AUROC for differentiating IBD from controls by NA: 0.827 (P < 0.001). Positive Correlation: NA with serum CRP (r = 0.376, P < 0.01) and fecal calprotectin (FC) (r = 0.575, P < 0.001). AUROC for differentiating FC < 120 µg/g from FC ≥ 120 µg/g by NA: 0.856 (P < 0.001). Medication Influence: Patients treated with corticosteroids (p = 0.022) or anti-IL-12/23 antibodies (p = 0.014) had higher fecal NA levels. |
|  | Negative Predictive Value (%) | Not directly specified. |
| Methodological Quality | Control Group | 17 healthy controls (age and sex matched). |
|  | Blinding | Not specified (cross-sectional observational study). |
|  | Reported Limitations | Single fecal sample collection. Absence of sphingolipid quantification in feces. Descriptive study (does not establish causality). Does not distinguish between free and bound VLCFAs. The mouse colitis model may differ from chronic colitis in humans. |

**Table 8. Article 8 - Gut Feeling: Biomarkers and Biosensors’ Potential in Revolutionizing Inflammatory Bowel Disease (IBD) Diagnosis and Prognosis—A Comprehensive Review**

| Category | Variable | Description |
| --- | --- | --- |
| Study Identification | Lead Author | Teixeira, B. |
|  | Year of Publication | 2025 |
|  | Country | Portugal |
|  | Journal | Biosensors |
|  | Study Type | Comprehensive Review |
|  | Level of Evidence | Not directly applicable for review. Consolidates evidence from various primary studies. |
| Population | Sample Size | Not applicable (review of primary studies with varied populations) |
|  | Age (mean ± SD) | Not applicable (variable among reviewed studies) |
|  | Sex (%) | Not applicable (variable among reviewed studies) |
|  | IBD Type | Crohn’s Disease (CD), Ulcerative Colitis (UC) |
| Biomarkers | Biomarker Type | Proteins, antibodies, microRNAs (miRNAs), DNA, microbiota. |
|  | Biomarker Name | C-reactive Protein (CRP), Erythrocyte Sedimentation Rate (ESR), Leucine-Rich Alpha-2 Glycoprotein (LRG), p-ANCA, ASCA, Calprotectin, Calgranulin C (S100A12), Lactoferrin, Lipocalin-2 (LCN-2), miRNAs (e.g., miR-223), TNF-α. |
|  | Detection Method | ELISA, Immunoturbidimetry, Chemiluminescence, SWV (Square Wave Voltammetry), SERS (Surface-Enhanced Raman Spectroscopy), CRISPR/Cas12a, Electrochemical Impedance Spectroscopy (EIS). |
|  | Sample Type | Serum, plasma, feces, sweat, gingival crevicular fluid, gastrointestinal fluid. |
|  | Diagnostic Purpose | Diagnosis, monitoring, prognosis, prediction of therapeutic response, detection of active inflammation. |
| Results | Sensitivity (%) | CRP (serum): 77–82% for UC, 83–92% for CD. ESR: ~78%. LRG: 87.9–99.3% for UC, 68–96% for CD. p-ANCA: 31–34%. ASCA: 38–42%. Calprotectin (fecal): 88%. Calgranulin C (fecal): 96%. Lactoferrin (fecal): 82%. LCN-2 (fecal): 85.7% for CD, 82% for UC. Peptide-based biosensor for CRP: LOD of 0.7 ng/mL. Electrochemical immunoassay for CRP: LOD of 0.15 nM (17 ng/mL). SERS nanosensor for TNF-α: LOD of 173 pg/L. CRISPR/Cas12a for FC: LOD of 1 ng/mL. |
|  | Specificity (%) | CRP (serum): 32–40% for UC, 70–89% for CD. ESR: ~78%. LRG: 86.2–99.9% for UC, 87–97% for CD. p-ANCA: 96–98%. ASCA: 91–94%. Calprotectin (fecal): 80%. Calgranulin C (fecal): 92%. Lactoferrin (fecal): 95%. LCN-2 (fecal): 45.5% for CD, 80% for UC. |
|  | Positive Predictive Value (%) | Calprotectin (sweat): CP detection in sweat (0.1–10 µg/mL), LOD of 0.1 µg/mL, distinction between inflamed and non-inflamed states. 3x increase during flares in IBD. Headspace gas sensors: Distinction between IBS and IBD: Sn 76%, Sp 88%, accuracy 76%. |
|  | Negative Predictive Value (%) | Not directly specified. |
| Methodological Quality | Control Group | Healthy controls and patients with other conditions (e.g., IBS) included in the reviewed primary studies. |
|  | Blinding | Not directly applicable for review. |
|  | Reported Limitations | Lack of specificity and accuracy of traditional tests (CRP, ESR). Disadvantages of endoscopic techniques (invasiveness, cost, operator dependence). Disadvantages of other imaging techniques (invasiveness, radiation, cost). Most biosensors are in the research phase, with challenges in cost, scalability, and ease of use for generalized clinical application. |

**Table 9. Article 9 - Inflammatory Bowel Disease Biomarkers**

| Category | Variable | Description |
| --- | --- | --- |
| Study Identification | Lead Author | Liu, D. |
|  | Year of Publication | 2022 |
|  | Country | USA |
|  | Journal | Med Res Rev |
|  | Study Type | Comprehensive Review |
|  | Level of Evidence | Not directly applicable for review. Consolidates evidence from various primary studies. |
| Population | Sample Size | Not applicable (review of primary studies with varied populations) |
|  | Age (mean ± SD) | Not applicable (variable among reviewed studies) |
|  | Sex (%) | Not applicable (variable among reviewed studies) |
|  | IBD Type | Crohn’s Disease (CD), Ulcerative Colitis (UC) |
| Biomarkers | Biomarker Type | Serum, fecal, miRNAs. |
|  | Biomarker Name | C-reactive Protein (CRP), Erythrocyte Sedimentation Rate (ESR), Anti-Saccharomyces cerevisiae antibodies (ASCA), perinuclear anti-neutrophil cytoplasmic antibodies (p-ANCA), nitric oxide (NO), Tumor Necrosis Factor-alpha (TNF-α), Interleukin-10 (IL-10), Suppression of Tumorigenicity 2 (ST2), Tumor Necrosis Factor Alpha-Induced Protein 6 (TNFAIP6), Calprotectin, S100A12, Lactoferrin, Lipocalin-2/NGAL, Myeloperoxidase (MPO), Matrix Metalloproteinases (MMPs), Intestinal Alkaline Phosphatase (IAP), microRNAs (miRNAs). |
|  | Detection Method | ELISA, high-sensitivity Immuno-PCR, IIF (Indirect Immunofluorescence). |
|  | Sample Type | Serum, feces, mucosal tissues. |
|  | Diagnostic Purpose | Diagnosis, inflammation monitoring, differentiation between UC and CD, assessment of therapy efficacy. |
| Results | Sensitivity (%) | ASCA+/p-ANCA- (CD diagnosis): 67%. ASCA-/p-ANCA+ (UC diagnosis): 78%. NO (differentiate active/inactive UC): 100% (cut-off 17.4 µM). NO (differentiate active/inactive CD): 88% (cut-off 14 µM). Calprotectin (fecal) for IBD diagnosis: 85–94%. S100A12 (fecal) for IBD diagnosis: 96% (cut-off 10 µg/g). Lactoferrin (fecal) for UC diagnosis: 81%. Lactoferrin (fecal) for CD diagnosis: 82%. NGAL (fecal) for active IBD diagnosis: 94.7%. MMP-9 (fecal) for active UC diagnosis: 96%. |
|  | Specificity (%) | ASCA+/p-ANCA- (CD diagnosis): 78%. ASCA-/p-ANCA+ (UC diagnosis): 67%. NO (differentiate active/inactive UC): 100% (cut-off 17.4 µM). NO (differentiate active/inactive CD): 69% (cut-off 14 µM). Calprotectin (fecal) for IBD diagnosis: 67–88%. S100A12 (fecal) for IBD diagnosis: 92% (cut-off 10 µg/g). Lactoferrin (fecal) for UC diagnosis: 82%. Lactoferrin (fecal) for CD diagnosis: 71%. NGAL (fecal) for active IBD diagnosis: 95.7%. MMP-9 (fecal) for active UC diagnosis: 75%. |
|  | Positive Predictive Value (%) | Serum CRP: Median of 40 mg/L (CD) and 20 mg/L (UC) at diagnosis. Levels in patients who responded to infliximab decreased. Serum IL-10: Increased in active UC and CD (144+34 pg/ml and 132+32 pg/ml, respectively) vs controls (44±9–5 pg/ml), P<0.001. Serum ST2: Median of 54 pg/ml (UC), 64 pg/ml (CD), 31 pg/ml (controls). Positively correlated with endoscopic activity (UC and CD). Decrease from 174 to 87 pg/ml in UC responders. Serum TNFAIP6: Median of 5.8 ng/ml (UC), 5.4 ng/ml (CD), 2.4 ng/ml (controls). Correlated with ESR, CRP, TNF-α. Stronger correlation with UC Mayo score (r = 0.65) than CRP (r = 0.51). Fecal Calprotectin: ~34 µg/g (healthy), ~3200 µg/g (CD), ~1900 µg/g (UC). Decrease from 1200 to 130 µg/g in anti-TNF responders. Fecal S100A12: ~470 ng/ml (active CD), ~400 ng/ml (active UC), ~75 ng/ml (controls). In children: ~95 µg/g (IBD) vs ~0.7 µg/g (controls). Fecal Lactoferrin: ~1 µg/g (healthy), ~440 µg/g (CD), ~1100 µg/g (UC). Decrease from 105 to 3 µg/g in anti-TNF responders. Fecal NGAL: 6 µg/g (UC), 5 µg/g (CD) vs 0.3 µg/g (healthy). Fecal MPO: ~100 µg/g (UC), ~60 µg/g (CD) vs ~4 µg/g (healthy). Sn 89% vs CRP 24% for UC. Fecal MMP-9: Median of 1.5 ng/ml (active CD), 0.6 ng/ml (inactive CD); 6.2 ng/ml (active UC), 0.7 ng/ml (inactive UC). Sn 96% and Sp 75% for active UC. IAP (Intestinal Alkaline Phosphatase): In CD, 22% less in inflammation vs non-inflammation. In UC, 20% less. |
|  | Negative Predictive Value (%) | Not directly specified. |
| Methodological Quality | Control Group | Healthy controls were included in all reviewed primary studies. |
|  | Blinding | Not directly applicable for review. |
|  | Reported Limitations | Endoscopy and biopsy are invasive. Some biomarkers (e.g., ASCA, p-ANCA) have low sensitivity. Serum TNF-α levels difficult to measure. Most studies of ST2, TNFAIP6, MPO, MMPs, and IAP need additional validation. |

**Table 10. Article 10 - Emerging Imaging Biomarkers in Crohn Disease**

| Category | Variable | Description |
| --- | --- | --- |
| Study Identification | Lead Author | Bane, O. |
|  | Year of Publication | 2021 |
|  | Country | USA, United Kingdom |
|  | Journal | Top Magn Reson Imaging |
|  | Study Type | Comprehensive Review |
|  | Level of Evidence | Not directly applicable for review. Consolidates evidence from various primary studies. |
| Population | Sample Size | Not applicable (review of primary studies with varied populations) |
|  | Age (mean ± SD) | Not applicable (variable among reviewed studies) |
|  | Sex (%) | Not applicable (variable among reviewed studies) |
|  | IBD Type | Crohn’s Disease (CD) |
| Biomarkers | Biomarker Type | Quantitative Magnetic Resonance Imaging (MRI). |
|  | Biomarker Name | Diffusion-weighted MRI (DWI), Apparent Diffusion Coefficient (ADC), Dynamic contrast-enhanced MRI (DCE-MRI), Ktrans, Ve, Magnetization transfer MRI (MT-MRI), Magnetization Transfer Ratio (MTR), Motility MRI. |
|  | Detection Method | MRI (DWI, DCE-MRI, MT-MRI, Motility). |
|  | Sample Type | Intestinal images. |
|  | Diagnostic Purpose | Diagnosis, assessment of disease activity, identification of intestinal fibrosis, assessment of biological treatment response, prognosis. |
| Results | Sensitivity (%) | DWI for intestinal inflammation: 92.9% (meta-analysis of 12 studies). DWI hyperintensity in intermediate b (500–600 s/mm²): Predictor of endoscopic inflammation (AUROC 0.702). ADC + Ktrans (Oto et al., 2011): 95%. ADC (Buisson et al., 2013): 96%. Motility MRI (Kopylov et al., 2016): 93%. ADC (Li et al., 2017): 97.3%. Ktrans + Ve + PF + ADC (Hectors et al., 2019): 96.3%. MTR for fibrosis (Li et al., 2018): 91%. Motility MRI (Menys et al., 2018): 92.9%. |
|  | Specificity (%) | DWI for intestinal inflammation: 91% (meta-analysis of 12 studies). DWI hyperintensity in intermediate b (500–600 s/mm²): Predictor of endoscopic inflammation (AUROC 0.702). ADC + Ktrans (Oto et al., 2011): 95%. ADC (Buisson et al., 2013): 96%. Motility MRI (Kopylov et al., 2016): 93%. ADC (Li et al., 2017): 97.3%. Ktrans + Ve + PF + ADC (Hectors et al., 2019): 96.3%. MTR for fibrosis (Li et al., 2018): 92%. Motility MRI (Menys et al., 2018): 71%. |
|  | Positive Predictive Value (%) | MaRIA: A segment score ≤ 7 has high performance for detecting active inflammation. ADC: Strong negative correlation with SES-CD (r = -0.76 to -0.88) and moderate negative correlation with CDEIS (r = -0.4). Inverse correlation with MaRIA (r = -0.77). DCE-MRI: Ktrans and Ve elevated in inflamed vs. normal intestinal segments. Enhancement gain (%) identifies marked fibrosis (AUROC = 0.93 for cut-off >24%). Positive correlation of time to peak enhancement with CRP and fecal calprotectin. MT-MRI: MTR correlated strongly with histological fibrosis (r = 0.77, P < 0.0001). Discriminated mild/moderate from moderate/severe fibrosis with AUROC of 0.92. Motility MRI: Reduced motility in affected segments associated with inflammatory activity. ADC to predict remission after biologic (Buisson et al., 2016): AUROC = 0.703 (cut-off ADC < 1.96). TAC of DKtrans and DBV to predict response to FMT (Zhu et al., 2017): 0.948. |
|  | Negative Predictive Value (%) | Not directly specified. |
| Methodological Quality | Control Group | Primary studies included healthy controls or non-inflamed intestinal segments. |
|  | Blinding | Not directly applicable for review. |
|  | Reported Limitations | Long acquisition time for DWI and DCE-MRI. Need for gadolinium in DCE-MRI. Variability of ADC between imaging protocols. DCE-MRI's ability to distinguish between fibrosis and inflammation is limited. Radiomics and AI are in early stages. Most studies have small samples. |

**Table 11. Article 11 - Serum Biomarkers in Diagnosis and Clinical Management of Inflammatory Bowel Disease: Anything New on the Horizon?**

| Category | Variable | Description |
| --- | --- | --- |
| Study Identification | Lead Author | Ondriš, J. |
|  | Year of Publication | 2024 |
|  | Country | Slovakia |
|  | Journal | Folia Biologica (Praha) |
|  | Study Type | Narrative Review |
|  | Level of Evidence | Not directly applicable for review. Consolidates evidence from various primary studies. |
| Population | Sample Size | Not applicable (review of primary studies with varied populations) |
|  | Age (mean ± SD) | Not applicable (variable among reviewed studies) |
|  | Sex (%) | Not applicable (variable among reviewed studies) |
|  | IBD Type | Crohn’s Disease (CD), Ulcerative Colitis (UC) |
| Biomarkers | Biomarker Type | Serum, protein, peptide, autoantibodies, molecular (miRNA, lncRNA). |
|  | Biomarker Name | Serum calprotectin (SC), Leucine-Rich Alpha-2 Glycoprotein (LRG), anti-OmpC, anti-I2, anti-CBir1 flagellin, microRNAs (miRNA, lncRNA), adropin, galectins (galectin-3, galectin-9, M2BP), SAA, IL-6, IL-8, eotaxin-1, homocysteine, complement C3, α2-macroglobulin, calgranulin C (S100A12), cholinesterases, adipokines (leptin, ghrelin, resistin, adiponectin), nesfatin 1, type VI collagen remodeling biomarkers (C6Ma3, PRO-C65), elastin degradation biomarkers (ELM-12, ELP-3), free thiols. |
|  | Detection Method | ELISA, LFIA (POCT), LEIT (latex-enhanced immunoturbidimetry), NEPH (nephelometry), ECL (electrochemiluminescence), FCM (flow cytometry), HPLC, SDS-PAGE, MS, enzymatic tests, spectrophotometry. |
|  | Sample Type | Serum, plasma, whole blood. |
|  | Diagnostic Purpose | Diagnosis, assessment of clinical and endoscopic activity, prediction of relapse, monitoring of treatment response, assessment of extraintestinal manifestations. |
| Results | Sensitivity (%) | SC (predict clinical remission): 65.6% (cut-off 5.3 mg/ml). SC (predict mucosal healing): 61.9% (cut-off 4.8 mg/ml). SC (CD diagnosis): 87.3%. SC (UC diagnosis): 98.5%. SC (active UC): AUROC 0.85, Sn 83.3%, Sp 81.25% (cut-off 4.4 mg/dl). SC (IBD diagnosis): AUROC 0.99, cut-off 925 ng/ml, Sn 98%, Sp 92%. SC (IBD diagnosis, AUROC 0.877): Sn 79% (cut-off 5.8 x 10³ µg/l). LRG (UC diagnosis vs. CRP): 0.73 vs 0.63. LRG (UC diagnosis, endoscopy): P=0.01. LRG (CD diagnosis vs. CRP): 0.71 vs 0.64. LRG (detect intestinal ulcers in CD): Significantly higher Sn vs CRP/CDAI. LRG (predict endoscopic remission CD): Sn 93.3% (cut-off 8.9 µg/ml). |
|  | Specificity (%) | SC (predict clinical remission): 67.6% (cut-off 5.3 mg/ml). SC (predict mucosal healing): 80.9% (cut-off 4.8 mg/ml). SC (CD diagnosis): 89.7%. SC (UC diagnosis): 75.6%. SC (active UC): AUROC 0.85, Sn 83.3%, Sp 81.25% (cut-off 4.4 mg/dl). SC (IBD diagnosis): AUROC 0.99, cut-off 925 ng/ml, Sn 98%, Sp 92%. SC (IBD diagnosis, AUROC 0.877): Sp 82% (cut-off 5.8 x 10³ µg/l). LRG (predict endoscopic remission CD): Sp 83.3% (cut-off 8.9 µg/ml). |
|  | Positive Predictive Value (%) | SC (CD relapse prediction after infliximab withdrawal): HR: 3.191 (5,675 ng/ml). SC (UC): Correlates with endoscopic scores, symptoms, and CRP (r = 0.56, P = 0.01; r = 0.64, P = 0.003; r = 0.97, P < 0.0001). LRG (UC): Correlates with clinical and endoscopic activity (P < 0.0001). AUROC 0.80. LRG (CD): Correlates with clinical and endoscopic activity. AUROC 0.79. LRG (CD, intestinal ulcers): Significantly higher AUROC vs CDAI and CRP. LRG (predict endoscopic remission CD): PPV 96.6%. LRG (monitor adalimumab treatment): Levels decrease with clinical and endoscopic improvement (SES-CD: r = 0.391 to 0.697; MES: r = 0.534 to 0.335). Correlates better with endoscopic activity than CRP and FC. |
|  | Negative Predictive Value (%) | LRG (predict endoscopic remission CD): NPV 71.4%. |
| Methodological Quality | Control Group | Healthy controls were included in the reviewed primary studies. |
|  | Blinding | Not directly applicable for review. |
|  | Reported Limitations | Most studies are monocentric with small samples. Lack of longitudinal monitoring and consistent assessment of endoscopic activity in some studies. SC was not effective in predicting relapse. Evidence for LRG and FC is still insufficient. |

**Table 12. Article 12 - Exploring potential biomarkers and therapeutic targets in inflammatory bowel disease: insights from a mega-analysis approach**

| Category | Variable | Description |
| --- | --- | --- |
| Study Identification | Lead Author | Stemmer, E. |
|  | Year of Publication | 2024 |
|  | Country | Israel |
|  | Journal | Front. Immunol. |
|  | Study Type | Mega-analysis and Machine Learning |
|  | Level of Evidence | Level II-1 (Mega-analysis of observational studies) |
| Population | Sample Size | Total of 697 participants: 569 IBD patients (386 UC, 183 CD) and 128 non-IBD controls. Independent validation set (GSE193677): 60 UC (rectum), 60 CD (ileum), 120 non-IBD controls (60 rectum, 60 ileum). Blood sample dataset (GSE186507): 111 blood samples (UC and CD). |
|  | Age (mean ± SD) | Not specified in the provided study data. |
|  | Sex (%) | Not specified in the provided study data (metadata include equal sex representation). |
|  | IBD Type | Crohn’s Disease (CD), Ulcerative Colitis (UC) and general IBD. |
| Biomarkers | Biomarker Type | Gene expression (DEGs), lncRNAs, serum proteins. |
|  | Biomarker Name | DEGs (e.g., DUOX2, DUOXA2, SAA1, SAA2, MMP3, REG1A, CHI3L1, AQP9, AQP8, CLDN8, CA1, OTOP2, SLC26A2, HMGCS2, DPP10), lncRNAs (ENSG00000254645, ENSG00000285744, ENSG00000287626, MIR4435-2HG, ADGRG3, KCNJ15, S100A8, S100A9). |
|  | Detection Method | RNA-seq (uniformly reprocessed raw data), Ingenuity Pathway Analysis (IPA), Machine Learning (ML) (SVM, KNN, Random Forest, Extra Trees, XGBoost, Naive Bayes), Connectivity Map (CMap). |
|  | Sample Type | Intestinal biopsies (colon and ileum), blood samples. |
|  | Diagnostic Purpose | Differentiation between IBD/UC/CD and non-IBD controls, identification of therapeutic targets, identification of non-invasive biomarkers. |
| Results | Sensitivity (%) | Not directly specified in the results tables for sensitivity and specificity of each biomarker, but rather for ML model performance. |
|  | Specificity (%) | Not directly specified in the results tables. |
|  | Positive Predictive Value (%) | DEGs in IBD: 2099 DEGs identified (1387 upregulated, 712 downregulated) in IBD vs. controls. Top 10 genes include DUOX2, DUOXA2 (up) and CLDN8, CA1 (down). ML-selected genes: - UC vs. controls (10 genes): Random Forest and KNN achieved 0.947 and 0.933 accuracy, respectively. - CD vs. controls (9 genes): KNN had 0.9 accuracy, Naive Bayes 0.89. - IBD vs. controls (34 genes): Both Random Forest and KNN models achieved 0.9 accuracy. - AUROC in validation (GSE193677): UC vs. control: >0.9 for RF and KNN; CD vs. control: >0.85 for KNN; IBD vs. control: >0.85 for RF and KNN. Non-invasive biomarkers (blood): Of 34 ML-selected genes, 29 were available in the blood dataset. 12 genes showed significant upregulation (p < 0.01) in individuals with severe endoscopic IBD manifestations. - Genes with most significant differences in blood: ADGRG3, KCNJ15, AQP9, MIR4435-2HG, S100A8, S100A9. LncRNAs: Three lncRNAs (ENSG00000285744, ENSG00000287626, MIR4435-2HG) were identified in the ML analysis for IBD. |
|  | Negative Predictive Value (%) | Not directly specified. |
| Methodological Quality | Control Group | 128 non-IBD controls (95 large intestine, 33 small intestine) in the mega-analysis set. 120 non-IBD controls (60 rectum, 60 ileum) in the independent validation set. |
|  | Blinding | Not specified. Mega-analysis and independent validation minimize the risk of bias. |
|  | Reported Limitations | Difficulty distinguishing CD and UC due to tissue-specific effects (biopsy). Validation of non-invasive biomarkers requires further investigation. The search for inhibitors for AQP proteins is challenging. |

**Table 13. Article 13 - Machine Learning Modeling from Omics Data as Prospective Tool for Improvement of Inflammatory Bowel Disease Diagnosis and Clinical Classifications**

| Category | Variable | Description |
| --- | --- | --- |
| Study Identification | Lead Author | Stankovic, B. |
|  | Year of Publication | 2021 |
|  | Country | Serbia |
|  | Journal | Genes |
|  | Study Type | Comprehensive Review |
|  | Level of Evidence | Not directly applicable for review. Consolidates evidence from various primary studies. |
| Population | Sample Size | Not applicable (review of primary studies with varied populations) |
|  | Age (mean ± SD) | Not applicable (variable among reviewed studies) |
|  | Sex (%) | Not applicable (variable among reviewed studies) |
|  | IBD Type | Crohn’s Disease (CD), Ulcerative Colitis (UC), unclassified IBD (IBDU) |
| Biomarkers | Biomarker Type | Genomic data (SNPs, exomes), transcriptomic data (gene expression, miRNAs). |
|  | Biomarker Name | SNPs (e.g., NOD2, PTPN22), genes (e.g., IL10, XIAP, CARD9), miRNAs (e.g., miR-215). |
|  | Detection Method | Genome-Wide Association Studies (GWAS), Immunochip, RNAseq, microarray, Whole Exome Sequencing, qPCR. |
|  | Sample Type | Intestinal biopsies, PBMCs (peripheral blood mononuclear cells), whole blood samples, exomes. |
|  | Diagnostic Purpose | IBD risk prediction, distinction between subtypes (CD, UC), prioritization of risk genes, prediction of postoperative activity, classification of UC/controls, discrimination of CD/UC/normals, prediction of infliximab response. |
| Results | Sensitivity (%) | CD (Immunochip): AUC 0.86. UC (Immunochip): AUC 0.83. CD (exome): AUC 0.74–0.83. UC (expression array): AUPRC 1 for active UC. CD (PBMC, expression array): 93.7%. UC (PBMC, expression array): 93.7%. IBD (PBMC, miRNAs): 90%. |
|  | Specificity (%) | CD (Immunochip): AUC 0.86. UC (Immunochip): AUC 0.83. CD (exome): AUC 0.74–0.83. UC (expression array): AUPRC 1 for active UC. CD (PBMC, expression array): 93.7%. UC (PBMC, expression array): 93.7%. IBD (PBMC, miRNAs): 90%. |
|  | Positive Predictive Value (%) | SNPs (GWAS or Immunochip): IBD risk prediction (AUC up to 0.75 for CD, 0.70 for UC). Immunochip SNPs: Prediction of CD probability (AUC 0.8). Exomes: Distinction between CD and healthy controls (AUC 0.7–0.75, AUPRC 0.73–0.80). Transcriptomic genes (array and RNAseq): Prioritization of IBD risk genes (AUC 0.775–0.829). Whole transcriptome: Identification of markers predicting postoperative activity (92–93% correct estimates). Whole blood miRNAs: IBD/control distinction (AUC not specified, but good results). Genomic expression: Prediction of infliximab response in UC (0.79 accuracy). RNAseq and microarray: Identification of susceptibility genes and predictive model for UC (AUC 0.95, AUPRC 0.97). |
|  | Negative Predictive Value (%) | Not directly specified. |
| Methodological Quality | Control Group | Healthy controls were included in the reviewed primary studies. |
|  | Blinding | Not directly applicable for review. |
|  | Reported Limitations | Most ML studies filter variants, which may reduce the ability to identify all causal variants. High computational costs. High heterogeneity of disease phenotypes. Small sample sizes in some studies (e.g., CAGI). MiRNA models in blood/urine have small samples and lack independent validation. |

**Table 14. Article 14 - Extrachromosomal Circular DNA: An Emerging Potential Biomarker for Inflammatory Bowel Diseases?**

| Category | Variable | Description |
| --- | --- | --- |
| Study Identification | Lead Author | Petito, V. |
|  | Year of Publication | 2024 |
|  | Country | Italy, USA, Denmark |
|  | Journal | Genes |
|  | Study Type | Comprehensive Review |
|  | Level of Evidence | Not directly applicable for review. Consolidates evidence from various primary studies. |
| Population | Sample Size | Not applicable (review of primary studies with varied populations, including humans and animal models) |
|  | Age (mean ± SD) | Not applicable (variable among reviewed studies) |
|  | Sex (%) | Not applicable (variable among reviewed studies) |
|  | IBD Type | Crohn’s Disease (CD), Ulcerative Colitis (UC) |
| Biomarkers | Biomarker Type | Extrachromosomal circular DNA (eccDNA) |
|  | Biomarker Name | eccDNA, cfDNA (circulating cell-free DNA), cf-mtDNA (circulating cell-free mitochondrial DNA), cf-ncDNA (circulating cell-free nuclear DNA). |
|  | Detection Method | Not specified (review of concepts and studies). Primary studies may use sequencing. |
|  | Sample Type | Plasma, serum, urine, intestinal biopsies (in animal models), biological fluid, lymphoid cells, fibroblasts, granulocytes. |
|  | Diagnostic Purpose | Identification of systemic and tissue inflammation, monitoring of disease severity, prediction of treatment response, screening for colitis-associated colorectal cancer (CAC). |
| Results | Sensitivity (%) | eccDNA: Increased levels have been demonstrated in UC patients. A study in mice showed a progressive increase in total cfDNA levels in DSS-induced colitis models, correlating with disease activity indicators. eccDNA: EccDNA activates innate immunity through the activation of DNA sensing pathways (STING and AIM2), inducing the production of IFN-1α, IFN-1β, IL-6, and TNF-α. |
|  | Specificity (%) | Not directly specified. |
|  | Positive Predictive Value (%) | Plasma eccDNA: Healthy patients had significantly longer eccDNA sequences in plasma than patients with gouty arthritis (Pang et al., 2022). cfDNA in UC: Pioneer study demonstrated the presence of cfDNA with microsatellite alteration in serum of UC patients. cfDNA in DSS-induced colitis (murine models): Significant increase in plasma cfDNA concentration. cfDNA in IBD: Patients with active IBD showed higher levels of total cfDNA and circulating cf-mtDNA. eccDNA in inflammation: Elevated eccDNA levels resulting from inflammatory processes characterized by cell death, accumulation of reactive oxygen species, and genomic instability. eccDNA as biomarker: Stability in tissues and blood is promising for integration as a prognostic biomarker. |
|  | Negative Predictive Value (%) | Not directly specified. |
| Methodological Quality | Control Group | Healthy controls were included in the reviewed primary studies. |
|  | Blinding | Not directly applicable for review. |
|  | Reported Limitations | Few clinical publications have specifically focused on eccDNA in IBD. Most studies on cfDNA in IBD did not distinguish eccDNA from other types of cfDNA. Research on eccDNA in IBD is still nascent. |

**Table 15. Article 15 - The Contribution of Genetic and Epigenetic Factors: An Emerging Concept in the Assessment and Prognosis of Inflammatory Bowel Diseases**

| Category | Variable | Description |
| --- | --- | --- |
| Study Identification | Lead Author | Minea, H. |
|  | Year of Publication | 2024 |
|  | Country | Romania |
|  | Journal | Int. J. Mol. Sci. |
|  | Study Type | Comprehensive Review |
|  | Level of Evidence | Not directly applicable for review. Consolidates evidence from various primary studies. |
| Population | Sample Size | Not applicable (review of primary studies with varied populations, including humans and animal models) |
|  | Age (mean ± SD) | Not applicable (variable among reviewed studies) |
|  | Sex (%) | Not applicable (variable among reviewed studies) |
|  | IBD Type | Crohn’s Disease (CD), Ulcerative Colitis (UC) |
| Biomarkers | Biomarker Type | Genetic (genes, SNPs), epigenetic (DNA methylation, histone modifications, lncRNAs, miRNAs). |
|  | Biomarker Name | Genes: NOD2, ATG16L1, IRGM, LRRK2, CARD9, CDH1, GNA12, PTPN2, HNF4-α, TRL-2, IL-23R, IL-17R, TNFSF15, FUT2, HLA-DQA105, HLA-DRB10103, HLA-DRB11501, HLA-DRB107, TPMT, NUDT15, CASP9, C1orf106, IL1RN, IL-10, ADAM17, SLCO1C1, IL-6, TNF-α, IL-17. SNPs: rs2066845 (NOD2), rs2647087 (HLA), rs1800629 (TNF-α), rs1799724 (TNF-α), rs767455 (TNF-α), rs1061624 (TNF-α), rs976881 (TNF-α), rs4149570 (TLR4), rs361525 (IL-6), rs3397 (IL-1), rs7576459 (IGFBP1/3), rs766748 (IL17F), rs1883136 (TRAF3IP2), rs755622 (MIF), rs3740691 (ARFGAP2), rs2275913 (IL17A). LncRNAs: TAP1, TESPA1, RPTOR, RPS6KA2, VMP1, TNSF10, FKBP5, BCL3, EYA4, SLIT2, FLI1, USP44, SND1, CND1, OL4A2, HDAC, GLI2, AXIN2, ABL1, TGFβ1, Setd2, SETDB1, KIF9-AS1, LINC01272, DIO3OS, ANRIL, RP11–679B19.1. miRNAs: miR-223, miR-23a, miR-155, miR-21, miR-10a, miR-200b, miR-675, miR-122a, miR-191a, miR-665, miR-20a, miR106b, miR-93, miR-16, miR-143, miR-132. |
|  | Detection Method | GWAS (Genome-Wide Association Studies), sequencing, qRT-PCR, EWAS (Epigenome-Wide Association Studies), microarray. |
|  | Sample Type | Blood, serum, intestinal biopsies, feces, cell lines. |
|  | Diagnostic Purpose | IBD risk prediction, risk stratification, prognosis, prediction of disease course, prediction of therapeutic response, prediction of adverse event occurrence. |
| Results | Sensitivity (%) | HLA-DQA102:01 and HLA-DRB107:01 haplotypes (thiopurine pancreatitis): Increased risk. CASP9 genes (rs1052571): OR 2.40 (95% CI 1.12–5.34), P=0.0224. C1orf106 (rs442905): IFX levels decreased in GA carriers (β = -0.949, P=0.025). IL1RN (rs3213448): IFX levels increased in GA carriers (OR 2.14, 95% CI 0.99–4.77). TLR4 (rs5030728): IFX levels decreased in GG vs GA+AA (OR 3.434, 95% CI 1.35–8.71, P=0.020). IL-10 (rs3024505): IFX levels increased. IL-23R (rs10489629): Protective effect against IFX failure (75% lower risk). IL-17 (rs766748): G/G genotype increases response (OR 5.123, 95% CI 1.261–27.77, P=0.0213). ATG16L1 (rs10210302): C/T and T/T genotype increases response to ADA (OR 9.44, 95% CI 2.49–35.83). DNA methylation (RPS6KA2/VMP1 for CD): AUC = 0.84. DNA methylation (TGFβ1 for UC): AUC = 0.99. lncRNA ANRIL (differentiates CD from controls): AUC = 0.803, Sn 86.1%, Sp 64.2%. lncRNA ANRIL (differentiates active phase/remission): AUC = 0.839, Sn 85.7%, Sp 71.2%. miRNA miR-146b-5p (for IBD): AUC = 0.869. |
|  | Specificity (%) | lncRNA ANRIL (differentiates CD from controls): Sp 64.2%. lncRNA ANRIL (differentiates active phase/remission): Sp 71.2%. miRNA miR-146b-5p (for IBD): AUC = 0.869. |
|  | Positive Predictive Value (%) | NOD2 SNPs (R702W, G908R, L1007fs): Increase CD risk by 15–40x. HLA-DQA102:01 and HLA-DRB107:01 SNPs: Pancreatitis risk 9% (heterozygotes) and 17% (homozygotes) after thiopurine. TNFRSF1A, IL1RN, IL18 SNPs: Benefit from anti-TNF. rs7576459 (IGFBP1/IGFBP3): Aggressive CD phenotype (OR 3.91, 95% CI 3.10–5.01, P < 0.001). CpG methylation on CDH1: Increased in inflamed ileal mucosa. LncRNAs (DIO3OS, KIF9-AS1, LINC01272): Increased in IBD, biomarkers for complications (e.g., RP11–679B19.1 for recurrent stricturing CD, OR 4.13). miRNAs (e.g., miR-223, miR-155): Inhibit inflammation or promote healing. |
|  | Negative Predictive Value (%) | Not directly specified. |
| Methodological Quality | Control Group | Healthy controls were included in the reviewed primary studies. |
|  | Blinding | Not directly applicable for review. |
|  | Reported Limitations | Low rate of family history of IBD. Some risk alleles (e.g., NOD2) may occur in healthy individuals. Most identified risk alleles contribute little to total disease variation. Evaluation of genotyping impact on therapeutic strategies is limited. Non-specificity of non-IBD cells in blood/tissue samples for epigenetic studies. Inaccuracies in GWAS due to linkage disequilibrium. |

**Table 16. Article 16 - Fecal Arachidonic Acid: A Potential Biomarker for Inflammatory Bowel Disease Severity**

| Category | Variable | Description |
| --- | --- | --- |
| Study Identification | Lead Author | Huss, M. |
|  | Year of Publication | 2025 |
|  | Country | Germany |
|  | Journal | Int. J. Mol. Sci. |
|  | Study Type | Cross-Sectional Observational Study (Clinical) |
|  | Level of Evidence | Level II-2 (Cohort study, no randomization) |
| Population | Sample Size | 62 IBD patients (38 CD, 24 UC) and 17 healthy controls. |
|  | Age (mean ± SD) | IBD: 42 years (range 19-78); Controls: 48 years (range 23-78). No significant difference between groups. |
|  | Sex (%) | IBD: 28/34 (female/male); Controls: 10/7 (female/male). No significant difference between groups. |
|  | IBD Type | Crohn’s Disease (CD), Ulcerative Colitis (UC) |
| Biomarkers | Biomarker Type | Fecal fatty acids (n-6 polyunsaturated). |
|  | Biomarker Name | Dihomo-γ-linolenic Acid (DGLA), Arachidonic Acid (AA), Adrenic Acid (AdA), Oleic Acid, Palmitic Acid, Stearic Acid, Linoleic Acid, Myristic Acid. |
|  | Detection Method | Gas Chromatography coupled with Mass Spectrometry (GC-MS). |
|  | Sample Type | Feces. |
|  | Diagnostic Purpose | Biomarker for IBD severity, IBD diagnosis, monitoring of disease activity. |
| Results | Sensitivity (%) | DGLA to differentiate IBD from controls: AUROC 0.769, P = 0.022. AA to differentiate IBD from controls: AUROC 0.859, P < 0.001. AdA to differentiate IBD from controls: AUROC 0.841, P < 0.001. AA to differentiate FC < 120 µg/g from FC ≥ 120 µg/g: 72% (cut-off 1.63 µmol/g). AdA to differentiate FC < 120 µg/g from FC ≥ 120 µg/g: 67% (cut-off 0.46 µmol/g). |
|  | Specificity (%) | DGLA to differentiate IBD from controls: AUROC 0.769, P = 0.022. AA to differentiate IBD from controls: AUROC 0.859, P < 0.001. AdA to differentiate IBD from controls: AUROC 0.841, P < 0.001. AA to differentiate FC < 120 µg/g from FC ≥ 120 µg/g: 77% (cut-off 1.63 µmol/g). AdA to differentiate FC < 120 µg/g from FC ≥ 120 µg/g: 84% (cut-off 0.46 µmol/g). |
|  | Positive Predictive Value (%) | AA, DGLA, AdA: Significantly elevated in IBD patients vs. controls. AA (r = 0.507, P < 0.001) and AdA (r = 0.480, P < 0.01): Positively correlated with fecal calprotectin (FC). Normal precursor levels: Linoleic acid and γ-linolenic acid (AA precursors) remained within normal ranges. AA and AdA: Correlated with elevated FC (>150 µg/g and >500 µg/g), but not with serum CRP. AA (P=0.017) and AdA (P=0.012): Higher levels in patients with calprotectin > 150 µg/g, and significantly elevated in those with >500 µg/g vs. patients with low FC (<50 µg/g). |
|  | Negative Predictive Value (%) | Not directly specified. |
| Methodological Quality | Control Group | 17 healthy controls. |
|  | Blinding | Not specified (cross-sectional observational study). |
|  | Reported Limitations | Small patient and control sample. Absence of lipid metabolite analysis. Serum fatty acid levels were not determined. Monocentric study, limiting the generalizability of results. IBD clinical scores were not included. Incomplete data on controls (laboratory values, BMI). |

**Table 17. Article 17 - Low-Density Lipoprotein and High-Density Lipoprotein as Biomarkers for Inflammatory Bowel Disease Activity Assessment**

| Category | Variable | Description |
| --- | --- | --- |
| Study Identification | Lead Author | Zhao, X. |
|  | Year of Publication | 2025 |
|  | Country | China |
|  | Journal | International Journal of General Medicine |
|  | Study Type | Retrospective Study |
|  | Level of Evidence | Level II-2 (Cohort study, no randomization) |
| Population | Sample Size | 490 IBD patients (280 CD, 210 UC) and 200 healthy controls. |
|  | Age (mean ± SD) | CD: 28 years (IQR, 23–38); UC: 45 years (IQR, 33–53); Controls: 35 years (IQR, 31–43). |
|  | Sex (%) | CD: 72.1% male; UC: 62.9% male; Controls: 70.0% male. |
|  | IBD Type | Crohn’s Disease (CD), Ulcerative Colitis (UC) |
| Biomarkers | Biomarker Type | Serum lipoproteins. |
|  | Biomarker Name | Low-Density Lipoprotein (LDL), High-Density Lipoprotein (HDL), C-reactive Protein (CRP), Erythrocyte Sedimentation Rate (ESR), Platelet Count (PLT), Serum albumin. |
|  | Detection Method | Routine laboratory tests. |
|  | Sample Type | Serum. |
|  | Diagnostic Purpose | Assessment of IBD activity, distinction between moderate-severe IBD. |
| Results | Sensitivity (%) | LDL to predict moderate-severe UC: 65.65% (cut-off 2.32 mmol/L). HDL to predict moderate-severe UC: 46.56% (cut-off 0.95 mmol/L). CRP to predict moderate-severe UC: 60.31% (cut-off 3.36 mg/L). ESR to predict moderate-severe UC: 77.10% (cut-off 7.48 mg/L). PLT to predict moderate-severe UC: 64.12% (cut-off 231.00 x 10⁹/L). |
|  | Specificity (%) | LDL to predict moderate-severe UC: 56.96% (cut-off 2.32 mmol/L). HDL to predict moderate-severe UC: 87.34% (cut-off 0.95 mmol/L). CRP to predict moderate-severe UC: 79.75% (cut-off 3.36 mg/L). ESR to predict moderate-severe UC: 69.62% (cut-off 7.48 mg/L). PLT to predict moderate-severe UC: 63.29% (cut-off 231.00 x 10⁹/L). |
|  | Positive Predictive Value (%) | Serum LDL and HDL levels: Significantly lower in IBD patients vs. healthy controls (P < 0.001 for both). In CD: HDL negatively correlated with HBI (rs= -0.341, p<0.001) and CDEIS (rs= -0.304, p<0.001), CRP (rs= -0.557, p<0.001) and ESR (rs= -0.484, p<0.001). LDL not correlated. In UC: LDL and HDL negatively correlated with clinical Mayo and UCEIS scores, CRP and ESR (P < 0.001 for all). CD (predict moderate-severe activity): AUC of HDL = 0.617 (P=0.0005); AUC of CRP = 0.671 (P<0.0001); AUC of ESR = 0.691 (P<0.0001). HDL specificity (65.85%) > CRP (52.44%) and ESR (60.98%). UC (predict moderate-severe activity): AUC of LDL = 0.635 (P=0.0006); AUC of HDL = 0.692 (P<0.0001); AUC of CRP = 0.734 (P<0.0001); AUC of ESR = 0.786 (P<0.0001); AUC of PLT = 0.668 (P<0.0001). LDL sensitivity (65.65%) > CRP (60.31%). HDL specificity (87.34%) > CRP (79.75%). |
|  | Negative Predictive Value (%) | LDL to predict moderate-severe UC: 49.44%. HDL to predict moderate-severe UC: 49.29%. CRP to predict moderate-severe UC: 54.78%. ESR to predict moderate-severe UC: 64.29%. PLT to predict moderate-severe UC: 51.55%. |
| Methodological Quality | Control Group | 200 healthy controls. |
|  | Blinding | Not specified (retrospective study). |
|  | Reported Limitations | Retrospective study at a single tertiary center (selection and recall bias). Did not evaluate lipid profiles in patients outside the 18-65 age range. Did not evaluate the relationship with fecal calprotectin. Did not include cardiovascular disease risk. Did not evaluate the effect of other factors such as diet. |

**Table 18. Article 18 - The Role of Calprotectin in the Diagnosis and Treatment of Inflammatory Bowel Disease**

| Category | Variable | Description |
| --- | --- | --- |
| Study Identification | Lead Author | Wang, W. |
|  | Year of Publication | 2025 |
|  | Country | China |
|  | Journal | Int. J. Mol. Sci. |
|  | Study Type | Comprehensive Review |
|  | Level of Evidence | Not directly applicable for review. Consolidates evidence from various primary studies. |
| Population | Sample Size | Not applicable (review of primary studies with varied populations, including humans and animal models) |
|  | Age (mean ± SD) | Not applicable (variable among reviewed studies) |
|  | Sex (%) | Not applicable (variable among reviewed studies) |
|  | IBD Type | Crohn’s Disease (CD), Ulcerative Colitis (UC) |
| Biomarkers | Biomarker Type | Protein derived from immune cells. |
|  | Biomarker Name | Calprotectin (fecal - FC and serum - SC), C-reactive Protein (CRP), Lactoferrin (Lf), Myeloperoxidase (fMPO), Eosinophil Cationic Protein (ECP), Eosinophil Derived Neurotoxin (EDN), Lipocalin-2 (LCN-2), S100A12. |
|  | Detection Method | Not specified (review of concepts and studies). Primary studies may use ELISA. |
|  | Sample Type | Feces, serum. |
|  | Diagnostic Purpose | Diagnosis of IBD, differentiation of functional disorders, monitoring of disease activity, prediction of relapse, assessment of therapeutic efficacy, screening for other gastrointestinal diseases. |
| Results | Sensitivity (%) | FC to differentiate IBD from functional disorders: 91%. FC for IBD diagnosis: 80% (in one study with 139 patients), with 83.3% for UC. FC to predict relapse in mucosal healing: 96% (cut-off 153 µg/g). |
|  | Specificity (%) | FC to differentiate IBD from functional disorders: 90%. |
|  | Positive Predictive Value (%) | Calprotectin levels: Reflect disease severity. FC: Increased levels (>50 µg/g) correlate with disease severity. Levels below 50 µg/g indicate negativity for IBD in adults. Levels below 600 µg/g and absence of symptoms in children indicate low probability of IBD. FC in colitis models (mice): Elevated levels in colitis induced by altered circadian rhythm. FC and ultrasound: Significant correlation in pediatric intestinal inflammation assessment. FC vs Lf (in UC patients): FC slightly superior to Lf. FC vs CRP (in UC patients): IL-6 levels surpassed inactive UC in differentiating active vs inactive UC, and showed positive correlation with FC (p = 0.021). FC vs SC: FC fully detects markers of endoscopic and histological remission, while SC is valuable in distinguishing between remission and active UC. FC vs MPO: FC surpassed MPO activity in predicting CD and extent of inflammation. FC vs FMPM (fecal myeloperoxidase): FC surpassed MPO activity in predicting CD and extent of inflammation. |
|  | Negative Predictive Value (%) | Not directly specified. |
| Methodological Quality | Control Group | Healthy controls and patients with other gastrointestinal diseases (e.g., IBS) included in the reviewed primary studies. |
|  | Blinding | Not specified (review of concepts and studies). |
|  | Reported Limitations | Endoscopy is invasive. FC can be affected by drugs (NSAIDs, PPIs), impacting its specificity. Correlation between calprotectin and disease may disappear with some treatments. Lack of consensus on FC cut-off values. FC specificity may be compromised by other infections and colorectal cancer. |

**Table 19. Article 19 - Oxidative stress-related biomarkers as promising indicators of inflammatory bowel disease activity: A systematic review and meta-analysis**

| Category | Variable | Description |
| --- | --- | --- |
| Study Identification | Lead Author | Tratenšek, A. |
|  | Year of Publication | 2024 |
|  | Country | Slovenia |
|  | Journal | Redox Biology |
|  | Study Type | Systematic Review and Meta-analysis |
|  | Level of Evidence | Level I (Systematic review of observational studies) |
| Population | Sample Size | 54 studies (patients with active and inactive IBD, healthy controls). Patients with active IBD: 45 to 1297; inactive IBD: 5 to 160; Controls: 8 to 741. |
|  | Age (mean ± SD) | Not specified in the provided study data (variable among reviewed studies). |
|  | Sex (%) | Not specified in the provided study data (variable among reviewed studies). |
|  | IBD Type | Crohn’s Disease (CD), Ulcerative Colitis (UC) |
| Biomarkers | Biomarker Type | Oxidative stress-related (oxidative damage products, antioxidants). |
|  | Biomarker Name | Oxidative damage: 8-iso-prostaglandin F2α (8-iso-PGF2α), Advanced Oxidation Protein Products (AOPP), Malondialdehyde (MDA). Enzymes: Paraoxonase 1 (PON-1), Catalase (CAT), Glutathione Peroxidase (GPx), Superoxide Dismutase (SOD). Vitamins and carotenoids: β-carotene, Lycopene, Total carotenoids, Vitamin A, Vitamin C, β-Cryptoxanthin, Lutein and Zeaxanthin, α-carotene, Vitamin E. Proteins: Albumin, Transferrin, Free thiols (R–SH), Selenoprotein P. Others: Total Antioxidant Capacity (TAC), Total Bilirubin (TBIL), Selenium (Se), Zinc (Zn), Serum Uric Acid (SUA). |
|  | Detection Method | Varied analytical methods (spectrophotometry, enzymatic assays, HPLC). |
|  | Sample Type | Plasma, serum, erythrocytes, leukocytes. |
|  | Diagnostic Purpose | Identification of IBD, assessment of disease activity, detection of exacerbations, differentiation between active and inactive IBD. |
| Results | Sensitivity (%) | Not directly specified. |
|  | Specificity (%) | Not directly specified. |
|  | Positive Predictive Value (%) | Active IBD vs. healthy controls: - Oxidative damage: 8-iso-PGF2α (SMD 3.65, P = 0.03), AOPP (SMD 1.21, P < 0.001), MDA (SMD 1.20, P < 0.001) significantly elevated. MDA 1.85x higher in active IBD vs controls (95% CI 1.53–2.23). - Enzymes: PON-1 (SMD -1.01, P = 0.005) and CAT (SMD -0.75, P < 0.001) reduced. GPx (plasma/serum) (SMD 0.82, P = 0.02) increased. - Vitamins/Carotenoids: β-carotene (SMD -1.52, P < 0.001), Lycopene (SMD -1.33, P < 0.001), Total carotenoids (SMD -1.29, P < 0.001), Vit. A (SMD -1.13, P = 0.01), Vit. C (SMD -1.01, P < 0.001), β-Cryptoxanthin (SMD -0.98, P < 0.001), Lutein and zeaxanthin (SMD -0.82, P < 0.001), α-carotene (SMD -0.75, P = 0.02) significantly reduced. - Proteins: Albumin (SMD -1.20, P < 0.001), Transferrin (SMD -0.88, P < 0.001), Free thiols (R–SH) (SMD -0.79, P < 0.001) significantly reduced. - Others: TAC (SMD -1.35, P < 0.001), TBIL (SMD -1.16, P = 0.002), Selenium (SMD -1.08, P = 0.008) significantly reduced. Inactive IBD vs. healthy controls: - Oxidative damage: 8-iso-PGF2α (SMD 3.56, P = 0.04), MDA (SMD 0.55, P = 0.04), AOPP (SMD 0.35, P = 0.03) significantly increased. - Vitamins/Carotenoids: β-carotene (SMD -0.69, p < 0.001), Total carotenoids (SMD -0.66, p = 0.01), Lycopene (SMD -0.63, p < 0.001), Vit. A (SMD -0.55, p < 0.001), β-Cryptoxanthin (SMD -0.50, p = 0.01), Vit. C (SMD -0.47, p < 0.001) significantly reduced. - Proteins: Selenoprotein P (SMD -1.19, p = 0.01), albumin (SMD -0.56, p < 0.001), R–SH (SMD -0.55, p = 0.002), transferrin (SMD -0.32, p = 0.002) significantly reduced. - Others: TAC (SMD -0.70, p = 0.003), Selenium (SMD -0.65, p < 0.001), TBIL (SMD -0.56, p < 0.001) significantly reduced. Active CD vs. inactive CD: MDA (SMD 0.85, P < 0.001) increased; PON-1 (SMD -1.20, P < 0.001), CAT (SMD -0.50, P = 0.001), albumin (SMD -1.00, P = 0.002), transferrin (SMD -0.58, P < 0.001) reduced; Total carotenoids (SMD -1.27, P = 0.002), β-carotene (SMD -1.14, P = 0.02), α-carotene (SMD -0.86, P = 0.001) reduced; TAC (SMD -0.55, P = 0.002) reduced. Active UC vs. inactive UC: PON-1 (SMD -0.96, P < 0.001), erythrocyte GPx (SMD -0.78, P < 0.001), CAT (SMD -0.65, P = 0.002), albumin (SMD -0.74, P = 0.004), transferrin (SMD -0.72, P = 0.01), R–SH (SMD -0.76, P = 0.007) reduced; Lycopene (SMD -1.49, P < 0.001), Total carotenoids (SMD -0.99, P = 0.004), Lutein and Zeaxanthin (SMD -0.74, P = 0.02), β-Cryptoxanthin (SMD -0.74, P = 0.02), Vit. E (SMD -0.86, P = 0.01) reduced. |
|  | Negative Predictive Value (%) | Not directly specified. |
| Methodological Quality | Control Group | Healthy controls were included in all reviewed primary studies. |
|  | Blinding | Not directly applicable for review. |
|  | Reported Limitations | Heterogeneous studies (different IBD subtypes, analytical methods, activity parameters). Most markers are not routinely measured. Variability in sample collection and external factors (diet, exercise, medications). Lack of longitudinal studies. |

**Table 20. Article 20 - Inflammatory Bowel Disease from the Perspective of Newer Innate Immune System Biomarkers**

| Category | Variable | Description |
| --- | --- | --- |
| Study Identification | Lead Author | Tobi, M. |
|  | Year of Publication | 2025 |
|  | Country | USA |
|  | Journal | Gastrointest Disord (Basel) |
|  | Study Type | Prospective Longitudinal Observational Study |
|  | Level of Evidence | Level II-2 (Cohort study, no randomization) |
| Population | Sample Size | 2185 patients selected from 2243 (31 UC, 18 CD, 2136 controls) with increased colorectal cancer risk. |
|  | Age (mean ± SD) | Controls: 60.0 ± 12.3 years; UC: 62.1 ± 14.5 years; CD: 51.4 ± 13.6 years. CD age significantly different from controls (P < 0.013). |
|  | Sex (%) | Controls: 89.1% (M/F); UC: 83.9% (M/F); CD: 83.3% (M/F). |
|  | IBD Type | Crohn’s Disease (CD), Ulcerative Colitis (UC) |
| Biomarkers | Biomarker Type | Innate immune system. |
|  | Biomarker Name | FERAD ratio (serum ferritin/fecal p87), FEREFF ratio (ferritin/p87 effluent), p87 (immunohistochemistry - IHC and ELISA in tissue extracts), NLR ratio (neutrophil/lymphocyte). |
|  | Detection Method | ELISA (for fecal p87 and tissue extracts), Immunohistochemistry (IHC) (for p87 in fixed tissue), electronic medical records (for ferritin and NLR). |
|  | Sample Type | Feces, serum, colonic biopsies (six regions). |
|  | Diagnostic Purpose | Assessment of InImS in IBD, differentiation of IBD vs. controls, comparison of UC vs. CD, neoplasia screening. |
| Results | Sensitivity (%) | Not directly specified. |
|  | Specificity (%) | Not directly specified. |
|  | Positive Predictive Value (%) | FERAD ratio: Not significantly different between UC and CD (p = 0.6). Significantly lower in IBD vs. controls (UC vs. Cnt: p < 0.00000071; CD vs. Cnt: p < 3 × 10−6), indicating deficient InImS in IBD. p87 IHC: In UC, gradient from 1 (cecum) to 0.66 (ascending colon) to 0.2-0.3 (transverse/descending colon), and zero in rectosigmoid. In CD, highest value in the rectum. p87 ELISA (extracts): In UC, low values in left colon segments. In CD, decreasing gradient from proximal to distal segments. Ferritin levels: First collection (143.9 ± 167.6) and last collection (192.4 ± 248.3) with P=0.6. Significantly different between IBD and controls (p < 0.002). |
|  | Negative Predictive Value (%) | Not directly specified. |
| Methodological Quality | Control Group | 2136 patients without IBD diagnosis. |
|  | Blinding | Not specified (observational study). |
|  | Reported Limitations | No data collected on therapy or disease severity for most patients. Small number of IBD patients. p87 in IHC and ELISA shows variations in expression, especially in the transverse colon. |

**Table 21. Article 21 - Proteomic Analysis Identifies Three Reliable Biomarkers of Intestinal Inflammation in the Stools of Patients With Inflammatory Bowel Disease**

| Category | Variable | Description |
| --- | --- | --- |
| Study Identification | Lead Author | Vitali, R. |
|  | Year of Publication | 2023 |
|  | Country | Italy |
|  | Journal | Journal of Crohn's and Colitis |
|  | Study Type | Diagnostic Accuracy Study (2D-DIGE, MALDI-TOF/TOF MS, ELISA) |
|  | Level of Evidence | Level II-2 (Cohort study, no randomization) |
| Population | Sample Size | 117 IBD patients (57 CD, 60 UC) and 31 controls. (Initially, 3 active CD patients, 3 inactive CD patients and 3 controls for 2D-DIGE). |
|  | Age (mean ± SD) | IBD: 46 (21–76) years for CD; 49.6 (24–83) years for UC. Controls: 45.8 (24–85) years. |
|  | Sex (%) | IBD: 27/30 (M/F) for CD; 35/27 (M/F) for UC. Controls: 20/11 (M/F). |
|  | IBD Type | Crohn’s Disease (CD), Ulcerative Colitis (UC) |
| Biomarkers | Biomarker Type | Fecal proteins. |
|  | Biomarker Name | Chymotrypsin C, Gelsolin, Rho GDP-Dissociation Inhibitor 2 (RhoGDI2), Fecal Calprotectin (FC), S100/A9. |
|  | Detection Method | 2-Difference Gel Electrophoresis (2-DIGE), MALDI-TOF/TOF Mass Spectrometry (MS), ELISA. |
|  | Sample Type | Feces. |
|  | Diagnostic Purpose | Identification of intestinal inflammation, IBD diagnosis, monitoring of disease severity, differentiation of IBD vs. controls. |
| Results | Sensitivity (%) | FC (CD): AUC 0.824, Sn 86%. Chymotrypsin C (CD): AUC 0.838, Sn 89%. Gelsolin (CD): AUC 0.988, Sn 91%. RhoGDI2 (CD): AUC 1.000, Sn 100%. FC (UC): AUC 0.876, Sn 75%. Chymotrypsin C (UC): AUC 0.922, Sn 100%. Gelsolin (UC): AUC 0.913, Sn 88%. RhoGDI2 (UC): AUC 1.000, Sn 100%. |
|  | Specificity (%) | FC (CD): AUC 0.824, Sp 68%. Chymotrypsin C (CD): AUC 0.838, Sp 81%. Gelsolin (CD): AUC 0.988, Sp 100%. RhoGDI2 (CD): AUC 1.000, Sp 100%. FC (UC): AUC 0.876, Sp 90%. Chymotrypsin C (UC): AUC 0.922, Sp 88%. Gelsolin (UC): AUC 0.913, Sp 100%. RhoGDI2 (UC): AUC 1.000, Sp 100%. |
|  | Positive Predictive Value (%) | Differential expression (2-DIGE): Chymotrypsin C (119.6x increased in active CD), Gelsolin (28.6x increased in active CD), RhoGDI2 (21.2x increased in active CD) vs. controls. FC (32.2x increased in active CD) as positive control. Fecal levels (ELISA): Chymotrypsin C, Gelsolin, RhoGDI2 and FC significantly increased in feces of CD and UC patients vs. controls (P < 0.001 for all). Correlation with endoscopic activity (Spearman coefficient, P < 0.001 for all): - CD: Chymotrypsin C r = 0.64; Gelsolin r = 0.82; RhoGDI2 r = 0.64; FC r = 0.73. - UC: Chymotrypsin C r = 0.76; Gelsolin r = 0.75; RhoGDI2 r = 0.63; FC r = 0.67. Correlation with FC (Spearman coefficient): - CD: Chymotrypsin C r = 0.49 (P < 0.001); Gelsolin r = 0.60 (P < 0.001); RhoGDI2 r = 0.61 (P < 0.001). - UC: Chymotrypsin C r = 0.59 (P < 0.001); Gelsolin r = 0.44 (P < 0.001); RhoGDI2 r = 0.34 (P < 0.01). Thresholds for IBD (cut-off with Youden index): - CD: FC 43.5 µg/g; Chymotrypsin C 9.87 ng/ml; Gelsolin 182.78 pg/ml; RhoGDI2 45.31 ng/ml. - UC: FC 122 µg/g; Chymotrypsin C 13 ng/ml; Gelsolin 202.19 pg/ml; RhoGDI2 42.88 ng/ml. |
|  | Negative Predictive Value (%) | Not directly specified. |
| Methodological Quality | Control Group | 31 controls (asymptomatic with gastrointestinal symptoms without endoscopic or histological changes). |
|  | Blinding | Not specified. |
|  | Reported Limitations | Low number of patients for the initial 2D-DIGE phase (3 patients/group). The correlation of RhoGDI2 with AUC=1 may overestimate significance. Need for validation in larger, independent cohorts. |

**Table 22. Article 22 - Current and emerging biomarkers for ulcerative colitis**

| Category | Variable | Description |
| --- | --- | --- |
| Study Identification | Lead Author | Nowak, J.K. |
|  | Year of Publication | 2023 |
|  | Country | Poland, United Kingdom |
|  | Journal | Expert Review of Molecular Diagnostics |
|  | Study Type | Comprehensive Review |
|  | Level of Evidence | Not directly applicable for review. Consolidates evidence from various primary studies. |
| Population | Sample Size | Not applicable (review of primary studies with varied populations) |
|  | Age (mean ± SD) | Not applicable (variable among reviewed studies) |
|  | Sex (%) | Not applicable (variable among reviewed studies) |
|  | IBD Type | Ulcerative Colitis (UC) |
| Biomarkers | Biomarker Type | Fecal, serum, autoantibodies, proteins, enzymes, miRNAs, lncRNAs, DNA methylation, glycomics, metabolomics, microbiota. |
|  | Biomarker Name | Established: Fecal calprotectin (FC), C-reactive protein (CRP). Investigational: PR3-ANCA, anti-αvβ6 integrin autoantibodies, fecal myeloperoxidase, fecal M2 PK, eosinophil-derived neurotoxin, fecal leukocyte lipase, Leucine-rich alpha-2 glycoprotein (LRG1), trefoil factor 3 (TFF3), Serum Amyloid A (SAA), dipeptidyl peptidase (DPP-4), serum gelsolin, total globulin, oncostatin M (OSM), VICM (citrullinated and MMP-degraded vimentin), CD6, colony-stimulating factor 1 (CSF1), FGF19, SLAMF1, MMP10, IL10, IL17A, IL12B, CX3CL1, CXCL9, CXCL11, MCP-1, miRNAs, DNA methylation, glycomes (N-linked glycoproteins), metabolites (e.g., bile acids, lipoproteins), microbiota (e.g., Ruminococcus gnavus, Bacteroides fragilis, Bifidobacteria), PCR, neutrophil-lymphocyte ratio, monocytes, mitochondrial DNA. |
|  | Detection Method | ELISA, Immunohistochemistry, spectrometry, PEA (Proximity Extension Assay), microarray, qPCR, mass spectrometry, infrared, PET-CT, sequencing. |
|  | Sample Type | Feces, serum, plasma, mucosal biopsies (colon), intestinal biopsies, whole blood. |
|  | Diagnostic Purpose | Diagnosis of UC, assessment of endoscopic/histological activity, prediction of relapse, treatment monitoring, prognosis, preclinical screening, differentiation of UC vs. CD/IBS. |
| Results | Sensitivity (%) | FC: ≤250 μg/g for histological remission (OR = 5.54); >250 μg/g (P < 0.001) for inflammation. Superior to fecal immunochemical test. PR3-ANCA: 75% (cut-off ≥3.5 U/mL). UC diagnosis: 44.5%. Anti-αvβ6 integrin autoantibodies: UC diagnosis: 76.3% (in UC vs IBS); 92% (original study). In recent study: 0.99 (cohort 1), 0.96 (cohort 2). Fecal myeloperoxidase: Sn 82% (cut-off >8.75 µg/g), Sp 90% (cut-off >3.65 µg/g) for active UC. LRG1: Sn 75.9% (cut-off 10.8 µg/mL) for active UC. Mucosal healing (AUC = 0.759). TFF3: AUC = 0.988 for mucosal healing. SAA: Differentiates Mayo 2/3: AUC = 0.807. DPP-4: Active UC vs remission: AUC = 0.71. Serum gelsolin: Clinical remission (AUC = 0.874). Endoscopic remission (AUC = 0.835). Serum OSM: UC diagnosis: AUC = 0.945. Predicts non-response to anti-TNF. Plasma N-linked glycomes: Prediction of treatment escalation (HR = 30.83). Transcriptome (CD300A, KPNA4, IL1R2, ELAVL1): UC diagnosis: AUC = 0.96. MiR-1307 (CD4+ T cell): UC diagnosis: AUC = 0.69. Ruminococcus gnavus (fecal): Positively associated with colitis extent. Fecal Chymotrypsin C: AUC 0.76 (active UC vs control). Fecal Gelsolin: AUC 0.75 (active UC vs control). Fecal RhoGDI2: AUC 0.63 (active UC vs control). |
|  | Specificity (%) | FC: ≤250 μg/g for histological remission (OR = 5.54); >250 μg/g (P < 0.001) for inflammation. Superior to fecal immunochemical test. PR3-ANCA: 69% (cut-off ≥3.5 U/mL). UC diagnosis: 95.6%. Anti-αvβ6 integrin autoantibodies: UC diagnosis: 96% (in UC vs IBS); 94.8% (original study). In recent study: 0.99 (cohort 1), 0.96 (cohort 2). Fecal myeloperoxidase: Sp 88% (cut-off >8.75 µg/g), Sn 90% (cut-off >3.65 µg/g) for active UC. LRG1: Sp 77.8% (cut-off 10.8 µg/mL) for active UC. Mucosal healing (AUC = 0.759). SAA: Differentiates Mayo 2/3: AUC = 0.807. DPP-4: Active UC vs remission: AUC = 0.71. Serum gelsolin: Clinical remission (AUC = 0.874). Endoscopic remission (AUC = 0.835). Serum OSM: UC diagnosis: AUC = 0.945. Fecal Chymotrypsin C: AUC 0.76 (active UC vs control). Fecal Gelsolin: AUC 0.75 (active UC vs control). Fecal RhoGDI2: AUC 0.63 (active UC vs control). |
|  | Positive Predictive Value (%) | FC: ≤250 μg/g predicts histological remission in 1 year (OR = 5.54). Lower colectomy risk in 7 years (HR = 0.30). Correlates with patient-reported outcomes. PR3-ANCA: Elevated baseline predicts need for steroids (AUC = 0.759). Correlates with Mayo score. Anti-αvβ6 integrin autoantibodies: Can predict UC development up to 10 years before onset. FC vs CRP: CRP can predict steroid response and colectomy. LRG1: Identifies clinical remission and mucosal healing (AUC = 0.759 and 0.665), better in deep remission. TFF3: AUC = 0.988 for mucosal healing. SAA: Identifies Mayo 2/3 patients better than CRP (AUC = 0.807 vs 0.701). Serum OSM: Associated with new UC diagnosis (AUC = 0.945). Predicts non-response to anti-TNF (AUC = 0.737) and vedolizumab (AUC = 0.685). PEA (CD6 and CSF1): Improves precision in predicting treatment escalation to 85.1%. PEA (8-proteins): Differentiates UC from CD (AUC = 0.725). DNA methylation: Methylation models predict treatment escalation (HR = 5.19). Transcriptome (CD300A, KPNA4, IL1R2, ELAVL1): UC diagnosis (AUC = 0.96). Transcriptome (17-gene qPCR): Predicts shorter time to treatment escalation (HR = 3.12). CLEC5A/CDH2 (expression): Predicts need for treatment escalation (HR = 23.4). Plasma N-linked glycomes: Prediction of treatment escalation (HR = 30.83). Plasma metabolites: Reflect high endoscopic activity (77% accuracy). Prediction of 6-month prognosis (74% accuracy). Serum bile acids: Active UC vs remission (AUC = 0.874). |
|  | Negative Predictive Value (%) | Not directly specified. |
| Methodological Quality | Control Group | Healthy controls and patients with other conditions (e.g., IBS, CD, PSC) included in the reviewed primary studies. |
|  | Blinding | Not directly applicable for review. |
|  | Reported Limitations | FC and CRP have limitations in specificity and precision. Many studies lack external validation. Complexity of ML models and omics technologies may hinder clinical translation. Replication of miRNA results is challenging. |

**Table 23. Article 23 - Machine learning identifies novel blood protein predictors of penetrating and stricturing complications in newly diagnosed paediatric Crohn’s disease.**

| Category | Variable | Description |
| --- | --- | --- |
| Study Identification | Lead Author | Ungaro, R.C. |
|  | Year of Publication | 2021 |
|  | Country | USA |
|  | Journal | Aliment. Pharmacol. Ther. |
|  | Study Type | Machine Learning Study |
|  | Level of Evidence | Level II-2 (Cohort study with ML analysis) |
| Population | Sample Size | 164 pediatric Crohn’s Disease (CD) patients newly diagnosed (mean age: 12.5 years, 66% male, 85% Caucasian). |
|  | Age (mean ± SD) | 12.5 years. |
|  | Sex (%) | 66% male. |
|  | IBD Type | Pediatric Crohn’s Disease (CD) |
| Biomarkers | Biomarker Type | Blood proteins. |
|  | Biomarker Name | CCL17 (Chemokine (C-C motif) ligand 17), GDF15 (Growth Differentiation Factor 15), IL1RA (Interleukin 1 Receptor Antagonist), MMP9 (Matrix Metalloproteinase 9), VEGFA (Vascular Endothelial Growth Factor A), MMP1 (Matrix Metalloproteinase 1), IL1B (Interleukin 1 beta), OSM (Oncostatin M). |
|  | Detection Method | Machine Learning analysis, multiplex immunoassay (Luminex Platform). |
|  | Sample Type | Serum. |
|  | Diagnostic Purpose | Prediction of penetrating and stricturing complications. |
| Results | Sensitivity (%) | Not directly specified in the results tables for sensitivity and specificity of each biomarker, but rather for ML model performance. |
|  | Specificity (%) | Not directly specified in the results tables. |
|  | Positive Predictive Value (%) | ML Model: Identified 17 serum proteins as predictors of complications. The 5 most important proteins were CCL17, GDF15, IL1RA, MMP9, and VEGFA. Model Performance: The model achieved an AUC of 0.88 (95% CI: 0.78-0.98) for predicting penetrating and stricturing complications within 1 year. Increased Proteins: Levels of IL1B, MMP1, and MMP9 were higher in patients who developed complications. Decreased Proteins: Levels of VEGFA were lower in patients who developed complications. |
|  | Negative Predictive Value (%) | Not directly specified. |
| Methodological Quality | Control Group | There was no traditional healthy control group, but rather a cohort of newly diagnosed pediatric CD patients, followed for complication development. |
|  | Blinding | Not specified. |
|  | Reported Limitations | Selection bias may exist due to the use of a specific pediatric CD cohort. The number of complications may be relatively low, affecting statistical power. The biological interpretation of the 17 proteins requires functional validation. |

**Table 24. Article 24 - Clinical Utility of Combined Fecal and Blood Biomarkers for Early Prediction of Crohn’s Disease in Children.**

| Category | Variable | Description |
| --- | --- | --- |
| Study Identification | Lead Author | Wang, T. |
|  | Year of Publication | 2021 |
|  | Country | China |
|  | Journal | Front. Pediatr. |
|  | Study Type | Diagnostic Accuracy Study (Retrospective) |
|  | Level of Evidence | Level II-2 (Cohort study, no randomization) |
| Population | Sample Size | 165 children (74 CD, 49 UC, 42 other gastrointestinal diseases (GID), 102 healthy controls (HC)). |
|  | Age (mean ± SD) | Not specified in the provided study data. |
|  | Sex (%) | Not specified in the provided study data. |
|  | IBD Type | Crohn’s Disease (CD), Ulcerative Colitis (UC) |
| Biomarkers | Biomarker Type | Fecal and blood. |
|  | Biomarker Name | Fecal Calprotectin (FC), Serum Calprotectin (SC), C-reactive Protein (CRP). |
|  | Detection Method | Not specified in the provided study data. |
|  | Sample Type | Feces, serum. |
|  | Diagnostic Purpose | Early prediction of Crohn’s Disease (CD) in children, differentiation of IBD from other GID and healthy controls. |
| Results | Sensitivity (%) | For UC vs. HC: FC + SC achieved an AUC of 0.983 (Sn 98%, Sp 95%). For CD vs. HC: FC + SC + CRP achieved an AUC of 0.992 (Sn 98%, Sp 99%). |
|  | Specificity (%) | For UC vs. HC: FC + SC achieved an AUC of 0.983 (Sn 98%, Sp 95%). For CD vs. HC: FC + SC + CRP achieved an AUC of 0.992 (Sn 98%, Sp 99%). |
|  | Positive Predictive Value (%) | For UC vs. HC: The combination of FC and SC achieved an AUC of 0.983 (Sn 98%, Sp 95%). For CD vs. HC: The combination of FC, SC, and CRP achieved an AUC of 0.992 (Sn 98%, Sp 99%). Correlations: FC, SC, and CRP showed significant correlations with disease activity (p < 0.001). Differentiation: The combination of biomarkers was also effective in differentiating CD from other GID. |
|  | Negative Predictive Value (%) | Not directly specified. |
| Methodological Quality | Control Group | 102 healthy controls (HC). 42 patients with other gastrointestinal diseases (GID). |
|  | Blinding | Not specified (retrospective study). |
|  | Reported Limitations | Retrospective study. The exact age of the pediatric population is not detailed. |

**Table 25. Article 25 - Combined Use of Fecal Biomarkers in Inflammatory Bowel Diseases: Oncostatin M and Calprotectin**

| Category | Variable | Description |
| --- | --- | --- |
| Study Identification | Lead Author | Cao, Y. |
|  | Year of Publication | 2021 |
|  | Country | China |
|  | Journal | Journal of Inflammation Research |
|  | Study Type | Cross-Sectional and Prospective Cohort Study |
|  | Level of Evidence | Level II-2 (Cohort study, no randomization) |
| Population | Sample Size | Group 1: 236 IBD patients (145 CD, 91 UC), 50 disease controls (DC) and 32 healthy controls (HC). Group 2: 62 IBD patients newly initiating infliximab (56 CD, 6 UC). |
|  | Age (mean ± SD) | CD: 32 (25–44) years; UC: 39 (28–51) years; DC (group 1): 31 (27.8–42.5) years; HC: 31 (26–47.8) years. |
|  | Sex (%) | CD: 40% female; UC: 41.8% female; DC (group 1): 34% female; HC: 53.1% female. |
|  | IBD Type | Crohn’s Disease (CD), Ulcerative Colitis (UC) |
| Biomarkers | Biomarker Type | Fecal (proteins), blood (proteins). |
|  | Biomarker Name | Fecal Calprotectin (FC), Fecal Oncostatin M (OSM), C-reactive Protein (CRP), Albumin (ALB), White Blood Cell Count (WBC), Platelet Count (PLT), Erythrocyte Sedimentation Rate (ESR), Hemoglobin (HB), Total Bilirubin (T-Bili). |
|  | Detection Method | ELISA (FC), Sandwich chemiluminescent immunoassay (OSM). |
|  | Sample Type | Feces, blood. |
|  | Diagnostic Purpose | Diagnosis of IBD, assessment of clinical and endoscopic activity, prediction of infliximab response. |
| Results | Sensitivity (%) | OSM to predict therapeutic response (week 28): Sn 66.7%. FC to predict therapeutic response (week 28): Sn 77.8%. |
|  | Specificity (%) | OSM to predict therapeutic response (week 28): Sp 92.5%. FC to predict therapeutic response (week 28): Sp 86.8%. |
|  | Positive Predictive Value (%) | Fecal OSM and FC: Increased in IBD patients vs. HC (OSM: P=0.001; FC: P<0.001) and vs. DC (OSM: P=0.006; FC: P<0.001). Combination of OSM and FC for IBD diagnosis: AUC 0.93. Fecal OSM and FC: Positively correlated with clinical and endoscopic activity. Fecal OSM and FC: Higher levels in active IBD patients vs. remission (P<0.001). Mucosal healing: Patients with mucosal healing had lower levels of FC (P < 0.001) and OSM (P = 0.006). AUC of FC to identify mucosal healing: 0.921. AUC of OSM + FC to identify mucosal healing: 0.923. Prediction of non-response to infliximab (week 28): Non-responders had higher levels of OSM (P = 0.002) and FC (P < 0.001). AUC of OSM = 0.763 (cut-off 132.4 pg/mL). AUC of FC = 0.834 (cut-off 4972 µg/g). AUC of OSM + FC = 0.859. Combination of fecal and blood biomarkers: AUC = 0.919. Prediction of non-response to infliximab (week 52): Non-responders had higher levels of OSM (P = 0.027) and FC (P = 0.041). AUC of OSM = 0.638. AUC of FC = 0.661. Combination of fecal and blood biomarkers: AUC = 0.887. |
|  | Negative Predictive Value (%) | Not directly specified. |
| Methodological Quality | Control Group | 50 disease controls (DC) (Behcet’s D., IBS, intestinal polyposis, non-IBD enteritis) and 32 healthy controls (HC). |
|  | Blinding | Not specified (observational study). |
|  | Reported Limitations | Remission sample in Group 1 may affect the accuracy of fecal OSM results. No dynamic changes were observed in OSM and FC levels in monitoring disease activity or predicting therapeutic response. Small sample size may introduce analytical bias. |

**Table 26. Article 26 - Calprotectin: from biomarker to biological function**

| Category | Variable | Description |
| --- | --- | --- |
| Study Identification | Lead Author | Jukic, A. |
|  | Year of Publication | 2021 |
|  | Country | Austria |
|  | Journal | Gut |
|  | Study Type | Comprehensive Review |
|  | Level of Evidence | Not directly applicable for review. Consolidates evidence from various primary studies. |
| Population | Sample Size | Not applicable (review of primary studies with varied populations, including humans and animal models) |
|  | Age (mean ± SD) | Not applicable (variable among reviewed studies) |
|  | Sex (%) | Not applicable (variable among reviewed studies) |
|  | IBD Type | Crohn’s Disease (CD), Ulcerative Colitis (UC) |
| Biomarkers | Biomarker Type | Protein (S100A8 and S100A9). |
|  | Biomarker Name | Fecal Calprotectin (FC), Serum Calprotectin (SC). |
|  | Detection Method | Not specified (review of concepts and studies). Primary studies may use ELISA. |
|  | Sample Type | Feces, serum. |
|  | Diagnostic Purpose | Diagnosis of intestinal inflammation, differentiation of IBD from non-inflammatory diseases, assessment of disease activity, monitoring of therapeutic response, prediction of relapse. |
| Results | Sensitivity (%) | FC to identify intestinal inflammation: Sn 92.9%. FC to differentiate IBD from functional diseases: Sn not specified, but cut-off values between 100–200 µg/g allow differentiation. |
|  | Specificity (%) | FC to identify intestinal inflammation: Sp 91%. FC to differentiate IBD from functional diseases: Sp not specified, but cut-off values between 100–200 µg/g allow differentiation. |
|  | Positive Predictive Value (%) | FC: Concentration in healthy individuals ranges between ~10 and 50 µg/g. Physiologically elevated in infants under 4 years. FC: Concentrations ≤40 µg/g rule out IBD (≤1% probability of having IBD). FC: Values >600 µg/g are strongly associated with IBD. FC: Strongly correlates with clinical and endoscopic disease activity (e.g., UC: grade 0 to 4 endoscopic activity correlates with FC from ≤16 µg/g to ~611 µg/g). FC: Significantly decreased in remission. Values >150 µg/g generally associated with relapse. STRIDE-II recommendations: <150 µg/g as remission target, 150–250 µg/g "gray zone". SC: Shows heterogeneous results as a biomarker for IBD. It is elevated in various systemic inflammatory diseases. |
|  | Negative Predictive Value (%) | Not directly specified. |
| Methodological Quality | Control Group | Healthy controls were included in all reviewed primary studies. |
|  | Blinding | Not directly applicable for review. |
|  | Reported Limitations | Non-specificity of FC (elevated in bacterial/viral infections, diverticulitis, colorectal cancer, drug-induced enteropathy). FC specificity is relatively low. Variations in FC cut-off interpretation. Poor appreciation of calprotectin's biological functions by most clinicians. |

**Table 27. Article 27 - Gut Microbiota Serves as a Crucial Independent Biomarker in Inflammatory Bowel Disease (IBD)**

| Category | Variable | Description |
| --- | --- | --- |
| Study Identification | Lead Author | Sharma, B. |
|  | Year of Publication | 2025 |
|  | Country | USA |
|  | Journal | Int. J. Mol. Sci. |
|  | Study Type | Comprehensive Review |
|  | Level of Evidence | Not directly applicable for review. Consolidates evidence from various primary studies. |
| Population | Sample Size | Not applicable (review of primary studies with varied populations, including humans and animal models) |
|  | Age (mean ± SD) | Not applicable (variable among reviewed studies) |
|  | Sex (%) | Not applicable (variable among reviewed studies) |
|  | IBD Type | Crohn’s Disease (CD), Ulcerative Colitis (UC), unclassified IBD (IBD-U) |
| Biomarkers | Biomarker Type | Gut microbiota, metabolites, morphogens, glycosylation, proteins (e.g., PDPN). |
|  | Biomarker Name | Bacteroidetes, Firmicutes, Faecalibacterium prausnitzii, Escherichia coli, Enterococcus sp., Proteobacteria, Actinobacteria, Fusobacterium, Ruminococcus gnavus, Bacteroides fragilis, Clostridium spp., Shigella spp., Akkermansia muciniphila, Methanosphaera stadtmanae, Veillonella parvula, Bifidobacterium, Staphylococcus, Lactobacillus, short-chain fatty acids (SCFAs), secondary bile acids (e.g., deoxycholic acid), lipids (sphingolipids, triacylglycerols, tetrapyrroles), nitric oxide (NO), Reactive Oxygen Species (ROS), podoplanin (PDPN), Wnt/β-catenin, Sonic Hedgehog (Shh), Bone Morphogenetic Proteins (BMPs). |
|  | Detection Method | Metagenomic sequencing, glucose hydrogen breath test, metabolomic studies, IHC. |
|  | Sample Type | Feces, mucosal biopsies (ileum, colon), serum. |
|  | Diagnostic Purpose | Independent biomarker and modulator in IBD pathogenesis, diagnosis, prognosis, monitoring, therapeutic target. |
| Results | Sensitivity (%) | Not directly specified. |
|  | Specificity (%) | Not directly specified. |
|  | Positive Predictive Value (%) | Microbiota in IBD: - Diversity: Decreased in CD and UC. - Faecalibacterium prausnitzii: Decreased in CD and UC (except in some de novo pediatric cases). - Escherichia coli: Increased in CD and UC. - Enterococcus sp.: Increased in CD and IBD. - Proteobacteria and Actinobacteria: Increased in IBD. - Clostridium spp.: Decreased in CD. - Metabolomics: Reduced SCFAs and secondary bile acids. Increased pro-inflammatory metabolites (e.g., sphingolipids, bile acids). - Glycosylation: Alterations in sialylation and fucosylation (e.g., decreased ST6 and FUT2) in IBD, compromising the mucosal barrier and increasing inflammation. - Podoplanin (PDPN): Increased expression in inflamed tissues, contributing to lymphangiogenesis and immune cell transport. - Oxidative Stress: Dysbiotic microbiota contributes to redox imbalance, leading to ROS and nitric oxide, exacerbating inflammation and tissue damage. |
|  | Negative Predictive Value (%) | Not directly specified. |
| Methodological Quality | Control Group | Healthy controls were included in all reviewed primary studies. |
|  | Blinding | Not directly applicable for review. |
|  | Reported Limitations | Most microbiota studies only evaluate bacteria, limiting conclusions about fungi and viruses. Microbiota results may vary with sampling technique (fecal vs. mucosal). |
