# Supplementary material for: A scoping review on emerging biomarkers in inflammatory bowel disease: Towards precision medicine in diagnosis and therapeutic management
Source: PLoS One. 2026 Jul 13;21(7):e0353295. doi: 10.1371/journal.pone.0353295 (PMC13362126; doi:10.1371/journal.pone.0353295)
Supplement: Attachment — Submitted filename: Dear Dr.docx [file pone.0353295.s004.docx]

Dear Dr.

We sincerely thank you, the Academic Editor, and both reviewers for the careful evaluation of our manuscript and for the constructive suggestions. We have revised the manuscript extensively to improve clarity, consistency, and methodological transparency, and we appreciate the opportunity to strengthen the work.

**Response to the Academic Editor**

**Comment:** Based on the feedback, our preliminary assessment leans toward recommending major revisions, considering the concerns raised by both reviewers. Improve the manuscript for better clarity and understanding.

**Response:** We thank the Academic Editor for this guidance. We revised the manuscript to improve clarity, coherence, and methodological consistency throughout the text. Specifically, we corrected inconsistencies in the search strategy, PRISMA flow, and data availability statement, and we added a concise summary table of the main biomarker groups to improve readability and practical interpretation. We also expanded the discussion to include a more critical assessment of heterogeneity, clinical translation, and the limitations of machine learning-based approaches.

**Response to Reviewer #1**

**Comment:** Very good work. Minor revisions are suggested to further improve the clarity and practical relevance of the manuscript. Specifically, the inclusion of a concise summary table highlighting the key biomarkers would enhance readability, and a short critical discussion addressing the challenges of clinical translation and validation of machine learning based approaches would add value. Overall, the manuscript demonstrates good scientific quality and is suitable for acceptance following these minor revisions.

**Response:** We are grateful for this positive assessment and for the constructive suggestions. In response, we added a concise summary table that organizes the main biomarker groups, their principal applications, and their key limitations. We also strengthened the discussion by adding a more critical section on the limitations of clinical translation, inter-study heterogeneity, and the validation requirements for machine learning-based biomarker models. These changes were made to enhance readability and to better position the findings within the current translational context.

**Response to Reviewer #2**

**Comment 1: Mixed evidence base.** The eligibility criteria include original research alongside systematic and narrative reviews. In a scoping review, including secondary literature creates a high risk of circular reporting.

**Response:** We appreciate this important methodological concern. Our aim in this scoping review was to map the breadth of emerging biomarker evidence in IBD, including both primary studies and higher-level syntheses when they provided relevant context on biomarker categories, analytical trends, and translational barriers. However, we agree that the use of secondary literature may complicate interpretation if not clearly differentiated. To address this, we revised the manuscript to more explicitly distinguish primary evidence from review-level evidence in the Results and Discussion, and we clarified the scope of the synthesis so that review articles are presented as contextual sources rather than as substitutes for primary patient-level evidence. We also emphasize in the Discussion that future biomarker validation should rely on multicenter primary studies with standardized designs and outcomes.

**Comment 2: Unretrieved reports.** Fifteen of 46 reports sought for retrieval could not be obtained.

**Response:** We thank the reviewer for highlighting this point. We revised the wording to describe this step in a neutral and methodologically appropriate way. The manuscript now states that these reports could not be included in the full-text eligibility assessment because sufficient information was not available at the time of screening to confirm eligibility according to the predefined criteria. We agree that this step must be reported transparently, and we have adjusted the Results section accordingly.

**Comment 3: PRISMA flowchart math.** There is a discrepancy between 784 identified records, 350 duplicates removed, and 406 records screened. Another section mentions 351 articles.

**Response:** We fully agree that this inconsistency needed correction. We rechecked the selection flow and standardized the PRISMA reporting throughout the manuscript so that the same numbers are used consistently in the Abstract, Results, and flow diagram. The flow now reads as follows: After removing duplicates (n = 350), ineligible records (n = 5), and other reasons (n = 23), 406 unique records advanced to screening. Title and abstract evaluation led to the exclusion of 360 records not aligning with the review's scope, resulting in 46 reports potentially relevant for full-text analysis. Of the 46 reports sought for retrieval, 15 were not included because their full texts were not available for detailed eligibility assessment at the time of screening, despite being identified as potentially relevant records. The remaining 31 reports underwent rigorous full-text eligibility assessment. 4 were excluded for not sufficiently elaborating on aspects of substantial value. Finally, 27 studies were included, forming the basis for data synthesis.

**Comment 4: Database discrepancy.** The Methods describe three databases, while the Abstract and Results mention five.

**Response:** We thank the reviewer for identifying this inconsistency. We revised the Methods to state clearly that the search strategy was applied in five major databases: PubMed/MEDLINE, Scopus, Web of Science, Embase, and Google Scholar. We also aligned the Abstract and Results with this wording to ensure complete consistency across the manuscript.

**Comment 5: Data availability statement contradiction.** The manuscript claims all data are in the Supporting Information, while the submission form states some restrictions apply.

**Response:** We agree that the statements must be consistent. Because this is a scoping review and does not involve primary participant-level data, we revised the Data Availability Statement to indicate that the minimal anonymized dataset necessary to reproduce the review findings is provided in the Supporting Information. We also adjusted the language to avoid implying unavailable restricted data. This correction was applied to harmonize the manuscript with the submission information.

**Comment 6: Narrative overview rather than structured scoping review.** The discussion is enthusiastic and lacks critical assessment of heterogeneity and readiness for clinical use.

**Response:** We appreciate this constructive critique. We revised the Discussion to better reflect the purpose of a scoping review, namely mapping the range and nature of evidence rather than drawing premature clinical conclusions. The revised text now includes a more critical discussion of heterogeneity across studies, differences in biomarker platforms and clinical endpoints, and the need for external validation before routine clinical implementation. We also replaced overly optimistic language with more balanced interpretation.

**Additional Clarifications**

**Summary table.** In response to Reviewer #1, we added a concise summary table that groups biomarkers by category and highlights their applications and limitations. This table is intended to improve readability and support rapid interpretation of the evidence.

**Machine learning discussion.** We added a short critical paragraph on machine learning-based biomarkers, emphasizing the exploratory nature of most models, the risk of overfitting, and the need for multicenter validation and reproducibility.

**Funding statement.** We also clarified the role of the funder in the cover letter, stating that the funders had no role in study design, data collection and analysis, decision to publish, or preparation of the manuscript, as requested by the journal.

**Ethics and protocols.** As this is a scoping review based exclusively on published literature, no human participant data were collected, and no additional laboratory protocols were required.

We hope that the revised manuscript now addresses all concerns raised by the Academic Editor and reviewers. We thank you again for your time and thoughtful feedback.

Sincerely,
