# Supplementary material for: A scoping review on emerging biomarkers in inflammatory bowel disease: Towards precision medicine in diagnosis and therapeutic management
Source: PLoS One. 2026 Jul 13;21(7):e0353295. doi: 10.1371/journal.pone.0353295 (PMC13362126; doi:10.1371/journal.pone.0353295)
Supplement: Peer review history

# Peer review: Decision Letter 0

**DOI:** 10.1371/journal.pone.0353295.r001

**Authors:** Saurabh Gupta

15 Apr 2026

PONE-D-26-07568

A Scoping Review on Emerging Biomarkers in Inflammatory Bowel Disease: Towards Precision Medicine in Diagnosis and Therapeutic Management.

PLOS One

Dear Dr. Souza Menezes,

Thank you for submitting your manuscript to PLOS ONE. After careful consideration, we feel that it has merit but does not fully meet PLOS ONE’s publication criteria as it currently stands. Therefore, we invite you to submit a revised version of the manuscript that addresses the points raised during the review process.

Please submit your revised manuscript by May 30 2026 11:59PM. If you will need more time than this to complete your revisions, please reply to this message or contact the journal office at plosone@plos.org. When you're ready to submit your revision, log on to https://www.editorialmanager.com/pone/ and select the 'Submissions Needing Revision' folder to locate your manuscript file.

Please include the following items when submitting your revised manuscript:

- A letter that responds to each point raised by the academic editor and reviewer(s). You should upload this letter as a separate file labeled 'Response to Reviewers'.
- A marked-up copy of your manuscript that highlights changes made to the original version. You should upload this as a separate file labeled 'Revised Manuscript with Track Changes'.
- An unmarked version of your revised paper without tracked changes. You should upload this as a separate file labeled 'Manuscript'.

If you would like to make changes to your financial disclosure, please include your updated statement in your cover letter. Guidelines for resubmitting your figure files are available below the reviewer comments at the end of this letter.

If applicable, we recommend that you deposit your laboratory protocols in protocols.io to enhance the reproducibility of your results. Protocols.io assigns your protocol its own identifier (DOI) so that it can be cited independently in the future. For instructions see: https://journals.plos.org/plosone/s/submission-guidelines#loc-laboratory-protocols. Additionally, PLOS ONE offers an option for publishing peer-reviewed Lab Protocol articles, which describe protocols hosted on protocols.io. Read more information on sharing protocols at https://plos.org/protocols?utm_medium=editorial-email&utm_source=authorletters&utm_campaign=protocols.

As the corresponding author, your ORCID iD is verified in the submission system and will appear in the published article. PLOS supports the use of ORCID, and we encourage all coauthors to register for an ORCID iD and use it as well. Please encourage your coauthors to verify their ORCID iD within the submission system before final acceptance, as unverified ORCID iDs will not appear in the published article. *Only*  the individual author can complete the verification step; PLOS staff *cannot*  verify ORCID iDs on behalf of authors.

We look forward to receiving your revised manuscript.

Kind regards,

Saurabh Gupta, Ph.D.

Academic Editor

PLOS One

Journal Requirements:

When submitting your revision, we need you to address these additional requirements.

1. Please ensure that your manuscript meets PLOS ONE's style requirements, including those for file naming. The PLOS ONE style templates can be found at

https://journals.plos.org/plosone/s/file?id=wjVg/PLOSOne_formatting_sample_main_body.pdf and

https://journals.plos.org/plosone/s/file?id=ba62/PLOSOne_formatting_sample_title_authors_affiliations.pdf

2. Thank you for stating the following financial disclosure:

"This research was funded by the Coordenação de Aperfeiçoamento de Pessoal de Nível Superior (CAPES), Brazil."

Please state what role the funders took in the study.  If the funders had no role, please state: "The funders had no role in study design, data collection and analysis, decision to publish, or preparation of the manuscript."

If this statement is not correct you must amend it as needed.

Please include this amended Role of Funder statement in your cover letter; we will change the online submission form on your behalf.

3. We note that you have indicated that there are restrictions to data sharing for this study. PLOS only allows data to be available upon request if there are legal or ethical restrictions on sharing data publicly. For more information on unacceptable data access restrictions, please see http://journals.plos.org/plosone/s/data-availability#loc-unacceptable-data-access-restrictions.

Before we proceed with your manuscript, please address the following prompts:

a) If there are ethical or legal restrictions on sharing a de-identified data set, please explain them in detail (e.g., data contain potentially identifying or sensitive patient information, data are owned by a third-party organization, etc.) and who has imposed them (e.g., a Research Ethics Committee or Institutional Review Board, etc.). Please also provide contact information for a data access committee, ethics committee, or other institutional body to which data requests may be sent.

b) If there are no restrictions, please upload the minimal anonymized data set necessary to replicate your study findings to a stable, public repository and provide us with the relevant URLs, DOIs, or accession numbers. For a list of recommended repositories, please see

https://journals.plos.org/plosone/s/recommended-repositories. You also have the option of uploading the data as Supporting Information files, but we would recommend depositing data directly to a data repository if possible.

We will update your Data Availability statement on your behalf to reflect the information you provide.

4. If the reviewer comments include a recommendation to cite specific previously published works, please review and evaluate these publications to determine whether they are relevant and should be cited. There is no requirement to cite these works unless the editor has indicated otherwise.

Additional Editor Comments:

Based on the feedback, our preliminary assessment leans toward recommending major revisions, considering the concerns raised by both reviewers. Improve the manuscript for better clarity and understanding.

[Note: HTML markup is below. Please do not edit.]

Reviewers' comments:

Reviewer's Responses to Questions

**Comments to the Author**

1. Is the manuscript technically sound, and do the data support the conclusions?

The manuscript must describe a technically sound piece of scientific research with data that supports the conclusions. Experiments must have been conducted rigorously, with appropriate controls, replication, and sample sizes. The conclusions must be drawn appropriately based on the data presented.

Reviewer #1: Yes

Reviewer #2: No

**********

2. Has the statistical analysis been performed appropriately and rigorously?

Reviewer #1: Yes

Reviewer #2: N/A

**********

3. Have the authors made all data underlying the findings in their manuscript fully available?

The PLOS Data policy requires authors to make all data underlying the findings described in their manuscript fully available without restriction, with rare exception (please refer to the Data Availability Statement in the manuscript PDF file). The data should be provided as part of the manuscript or its supporting information, or deposited to a public repository. For example, in addition to summary statistics, the data points behind means, medians and variance measures should be available. If there are restrictions on publicly sharing data—e.g. participant privacy or use of data from a third party—those must be specified.

Reviewer #1: Yes

Reviewer #2: Yes

**********

4. Is the manuscript presented in an intelligible fashion and written in standard English?

PLOS ONE does not copyedit accepted manuscripts, so the language in submitted articles must be clear, correct, and unambiguous. Any typographical or grammatical errors should be corrected at revision, so please note any specific errors here.

Reviewer #1: Yes

Reviewer #2: Yes

**********

5. Review Comments to the Author

Please use the space provided to explain your answers to the questions above. You may also include additional comments for the author, including concerns about dual publication, research ethics, or publication ethics. (Please upload your review as an attachment if it exceeds 20,000 characters)

Reviewer #1: Greetings

Very good work dears.

Minor revisions are suggested to further improve the clarity and practical relevance of the manuscript. Specifically, the inclusion of a concise summary table highlighting the key biomarkers would enhance readability, and a short critical discussion addressing the challenges of clinical translation and validation of machine learning based approaches would add value. Overall, the manuscript demonstrates good scientific quality and is suitable for acceptance following these minor revisions.

Kind regards,

Reviewer #2: While the topic of emerging biomarkers in IBD toward precision medicine is of high clinical interest, the current manuscript suffers from significant methodological and reporting flaws that undermine the validity of its synthesis.

Methodological FlawsMixed Evidence Base: The eligibility criteria explicitly include original research alongside systematic and narrative reviews . In a scoping review, including secondary literature (which constitutes 51.9% of your included studies) creates a high risk of "circular reporting." This dilutes primary evidence and makes it impossible to conduct a rigorous mapping of unique patient data.

Unretrieved Reports: The authors report that 15 out of 46 (32.6%) reports sought for retrieval could not be obtained For a scoping review, this is an unacceptably high attrition rate that suggests an incomplete search or a lack of institutional access, which biases the final synthesis.

Reporting and Transparency Inconsistencies PRISMA Flowchart Math: There is a significant discrepancy in the search totals. The text states that 784 records were identified and 350 duplicates were removed .

Mathematically, this should result in 434 unique records, yet the manuscript claims 406 unique records advanced to screening. Furthermore, another section mentions 351 articles were identified after duplicate removal . These contradictions must be resolved.

Database Discrepancy: The Methodology section describes a strategy applied to three main databases , whereas the Abstract and Results sections claim five major databases were searched.Data Availability Statement: There is a contradiction between the "Additional Information" response ("No- some restrictions will apply" ) and the final manuscript text claiming all data is in the Supporting Information.

The paper reads as a broad narrative overview rather than a structured scoping review. A scoping review should map the range and nature of evidence. The discussion relies on enthusiastic summary language (e.g., "vast potential" ) rather than a critical assessment of how study heterogeneity which the authors admit is a major limitation impacts the readiness of these biomarkers for clinical use.

**********

6. PLOS authors have the option to publish the peer review history of their article (what does this mean?). If published, this will include your full peer review and any attached files.

If you choose “no”, your identity will remain anonymous but your review may still be made public.

**Do you want your identity to be public for this peer review?**  For information about this choice, including consent withdrawal, please see our Privacy Policy.

Reviewer #1: No

Reviewer #2: No

**********

[NOTE: If reviewer comments were submitted as an attachment file, they will be attached to this email and accessible via the submission site. Please log into your account, locate the manuscript record, and check for the action link "View Attachments". If this link does not appear, there are no attachment files.]

To ensure your figures meet our technical requirements, please review our figure guidelines: https://journals.plos.org/plosone/s/figures

You may also use PLOS’s free figure tool, NAAS, to help you prepare publication quality figures: https://journals.plos.org/plosone/s/figures#loc-tools-for-figure-preparation.

NAAS will assess whether your figures meet our technical requirements by comparing each figure against our figure specifications.

# Author comment: Author response to Decision Letter 1

**DOI:** 10.1371/journal.pone.0353295.r002

18 May 2026

Dear Dr.

We sincerely thank you, the Academic Editor, and both reviewers for the careful evaluation of our manuscript and for the constructive suggestions. We have revised the manuscript extensively to improve clarity, consistency, and methodological transparency, and we appreciate the opportunity to strengthen the work.

Response to the Academic Editor

Comment: Based on the feedback, our preliminary assessment leans toward recommending major revisions, considering the concerns raised by both reviewers. Improve the manuscript for better clarity and understanding.

Response: We thank the Academic Editor for this guidance. We revised the manuscript to improve clarity, coherence, and methodological consistency throughout the text. Specifically, we corrected inconsistencies in the search strategy, PRISMA flow, and data availability statement, and we added a concise summary table of the main biomarker groups to improve readability and practical interpretation. We also expanded the discussion to include a more critical assessment of heterogeneity, clinical translation, and the limitations of machine learning-based approaches.

Response to Reviewer #1

Comment: Very good work. Minor revisions are suggested to further improve the clarity and practical relevance of the manuscript. Specifically, the inclusion of a concise summary table highlighting the key biomarkers would enhance readability, and a short critical discussion addressing the challenges of clinical translation and validation of machine learning based approaches would add value. Overall, the manuscript demonstrates good scientific quality and is suitable for acceptance following these minor revisions.

Response: We are grateful for this positive assessment and for the constructive suggestions. In response, we added a concise summary table that organizes the main biomarker groups, their principal applications, and their key limitations. We also strengthened the discussion by adding a more critical section on the limitations of clinical translation, inter-study heterogeneity, and the validation requirements for machine learning-based biomarker models. These changes were made to enhance readability and to better position the findings within the current translational context.

Response to Reviewer #2

Comment 1: Mixed evidence base. The eligibility criteria include original research alongside systematic and narrative reviews. In a scoping review, including secondary literature creates a high risk of circular reporting.

Response: We appreciate this important methodological concern. Our aim in this scoping review was to map the breadth of emerging biomarker evidence in IBD, including both primary studies and higher-level syntheses when they provided relevant context on biomarker categories, analytical trends, and translational barriers. However, we agree that the use of secondary literature may complicate interpretation if not clearly differentiated. To address this, we revised the manuscript to more explicitly distinguish primary evidence from review-level evidence in the Results and Discussion, and we clarified the scope of the synthesis so that review articles are presented as contextual sources rather than as substitutes for primary patient-level evidence. We also emphasize in the Discussion that future biomarker validation should rely on multicenter primary studies with standardized designs and outcomes.

Comment 2: Unretrieved reports. Fifteen of 46 reports sought for retrieval could not be obtained.

Response: We thank the reviewer for highlighting this point. We revised the wording to describe this step in a neutral and methodologically appropriate way. The manuscript now states that these reports could not be included in the full-text eligibility assessment because sufficient information was not available at the time of screening to confirm eligibility according to the predefined criteria. We agree that this step must be reported transparently, and we have adjusted the Results section accordingly.

Comment 3: PRISMA flowchart math. There is a discrepancy between 784 identified records, 350 duplicates removed, and 406 records screened. Another section mentions 351 articles.

Response: We fully agree that this inconsistency needed correction. We rechecked the selection flow and standardized the PRISMA reporting throughout the manuscript so that the same numbers are used consistently in the Abstract, Results, and flow diagram. The flow now reads as follows: After removing duplicates (n = 350), ineligible records (n = 5), and other reasons (n = 23), 406 unique records advanced to screening. Title and abstract evaluation led to the exclusion of 360 records not aligning with the review's scope, resulting in 46 reports potentially relevant for full-text analysis. Of the 46 reports sought for retrieval, 15 were not included because their full texts were not available for detailed eligibility assessment at the time of screening, despite being identified as potentially relevant records. The remaining 31 reports underwent rigorous full-text eligibility assessment. 4 were excluded for not sufficiently elaborating on aspects of substantial value. Finally, 27 studies were included, forming the basis for data synthesis.

Comment 4: Database discrepancy. The Methods describe three databases, while the Abstract and Results mention five.

Response: We thank the reviewer for identifying this inconsistency. We revised the Methods to state clearly that the search strategy was applied in five major databases: PubMed/MEDLINE, Scopus, Web of Science, Embase, and Google Scholar. We also aligned the Abstract and Results with this wording to ensure complete consistency across the manuscript.

Comment 5: Data availability statement contradiction. The manuscript claims all data are in the Supporting Information, while the submission form states some restrictions apply.

Response: We agree that the statements must be consistent. Because this is a scoping review and does not involve primary participant-level data, we revised the Data Availability Statement to indicate that the minimal anonymized dataset necessary to reproduce the review findings is provided in the Supporting Information. We also adjusted the language to avoid implying unavailable restricted data. This correction was applied to harmonize the manuscript with the submission information.

Comment 6: Narrative overview rather than structured scoping review. The discussion is enthusiastic and lacks critical assessment of heterogeneity and readiness for clinical use.

Response: We appreciate this constructive critique. We revised the Discussion to better reflect the purpose of a scoping review, namely mapping the range and nature of evidence rather than drawing premature clinical conclusions. The revised text now includes a more critical discussion of heterogeneity across studies, differences in biomarker platforms and clinical endpoints, and the need for external validation before routine clinical implementation. We also replaced overly optimistic language with more balanced interpretation.

Additional Clarifications

Summary table. In response to Reviewer #1, we added a concise summary table that groups biomarkers by category and highlights their applications and limitations. This table is intended to improve readability and support rapid interpretation of the evidence.

Machine learning discussion. We added a short critical paragraph on machine learning-based biomarkers, emphasizing the exploratory nature of most models, the risk of overfitting, and the need for multicenter validation and reproducibility.

Funding statement. We also clarified the role of the funder in the cover letter, stating that the funders had no role in study design, data collection and analysis, decision to publish, or preparation of the manuscript, as requested by the journal.

Ethics and protocols. As this is a scoping review based exclusively on published literature, no human participant data were collected, and no additional laboratory protocols were required.

We hope that the revised manuscript now addresses all concerns raised by the Academic Editor and reviewers. We thank you again for your time and thoughtful feedback.

Sincerely,

Attachment Submitted filename: Dear Dr.docx

# Editor report: Decision Letter 1

**DOI:** 10.1371/journal.pone.0353295.r003

**Authors:** Saurabh Gupta, Saurabh Gupta

22 Jun 2026

A Scoping Review on Emerging Biomarkers in Inflammatory Bowel Disease: Towards Precision Medicine in Diagnosis and Therapeutic Management.

PONE-D-26-07568R1

Dear Dr. João Daniel,

We’re pleased to inform you that your manuscript has been judged scientifically suitable for publication and will be formally accepted for publication once it meets all outstanding technical requirements.

Within one week, you’ll receive an e-mail detailing the required amendments. When these have been addressed, you’ll receive a formal acceptance letter and your manuscript will be scheduled for publication.

An invoice will be generated when your article is formally accepted. Please note, if your institution has a publishing partnership with PLOS and your article meets the relevant criteria, all or part of your publication costs will be covered. Please make sure your user information is up-to-date by logging into Editorial Manager at Editorial Manager® and clicking the ‘Update My Information' link at the top of the page. For questions related to billing, please contact billing support.

If your institution or institutions have a press office, please notify them about your upcoming paper to help maximize its impact. If they’ll be preparing press materials, please inform our press team as soon as possible -- no later than 48 hours after receiving the formal acceptance. Your manuscript will remain under strict press embargo until 2 pm Eastern Time on the date of publication. For more information, please contact onepress@plos.org.

Kind regards,

Saurabh Gupta, Ph.D.

Academic Editor

PLOS One

Additional Editor Comments (optional):

The authors have satisfactorily addressed the comments raised by the reviewers and revised the manuscript accordingly.

Reviewers' comments:

# Editor report: Acceptance letter

**DOI:** 10.1371/journal.pone.0353295.r004

**Authors:** Saurabh Gupta, Saurabh Gupta

PONE-D-26-07568R1

PLOS One

Dear Dr. Souza Menezes,

I'm pleased to inform you that your manuscript has been deemed suitable for publication in PLOS One. Congratulations! Your manuscript is now being handed over to our production team.

At this stage, our production department will prepare your paper for publication. This includes ensuring the following:

* All references, tables, and figures are properly cited

* All relevant supporting information is included in the manuscript submission,

* There are no issues that prevent the paper from being properly typeset

You will receive further instructions from the production team, including instructions on how to review your proof when it is ready. Please keep in mind that we are working through a large volume of accepted articles, so please give us a few days to review your paper and let you know the next and final steps.

Lastly, if your institution or institutions have a press office, please let them know about your upcoming paper now to help maximize its impact. If they'll be preparing press materials, please inform our press team within the next 48 hours. Your manuscript will remain under strict press embargo until 2 pm Eastern Time on the date of publication. For more information, please contact onepress@plos.org.

You will receive an invoice from PLOS for your publication fee after your manuscript has reached the completed accept phase. If you receive an email requesting payment before acceptance or for any other service, this may be a phishing scheme. Learn how to identify phishing emails and protect your accounts at https://explore.plos.org/phishing.

If we can help with anything else, please email us at customercare@plos.org.

Thank you for submitting your work to PLOS ONE and supporting open access.

Kind regards,

PLOS ONE Editorial Office Staff

on behalf of

Dr. Saurabh Gupta

Academic Editor

PLOS One
